# BioSounds: an open-source, online platform for ecoacoustics

**DOI:** 10.12688/f1000research.26369.1

**Published:** 2020-10-12

**Authors:** Kevin Darras, Noemí Pérez, Mauladi, Tara Hanf-Dressler

**Affiliations:** 1 Agroecology, University of Göttingen, Göttingen, Niedersachsen, 37077, Germany; 2 Department of Information Systems, Universitas Jambi, Jambi, Jambi, 36122, Indonesia

**Keywords:** Soundscape, sound analysis, ecoacoustics, passive acoustic monitoring, automated sound recording, autonomous recording units, spectrogram, audio annotation

## Abstract

Passive acoustic monitoring of soundscapes and biodiversity produces vast amounts of audio recordings. However, the management of these raw data presents technical challenges and their analysis suffers from bottlenecks. A multitude of software solutions exist, but none can perform all the data processing needed by ecologists for analysing large acoustic data sets. The field of ecoacoustics needs a software tool that is free, evolving, and accessible. We take a step in that direction and present BioSounds: an open-source, online platform for ecoacoustics designed by ecologists and built by software engineers. Biosounds can be used for archiving and sharing recordings, manually creating and reviewing annotations of sonant animals in soundscapes, analysing audio in time and frequency, and storing reference recordings for different taxa. We present its features and structure, and compare it with similar software. We describe its operation mode and the workflow for typical use cases such as the analysis of bird and bat communities sampled in soundscape recordings. BioSounds is available from: https://github.com/nperezg/biosounds

## Introduction

Automated, passive recording for biodiversity research has come of age. It presents new opportunities for ecologists, but yields huge amounts of data that are challenging to manage ^1^. The resulting recordings are raw data that require considerable effort to extract the ecological information contained within. To realise the potential of ecoacoustics projects, different software tools are required in different data processing and analysis stages: First, soundscape recordings - comprising all sounds recorded in a landscape ^2^ - need to be archived and made accessible to collaborators, either locally or remotely ^3^. Sometimes, an optional pre-processing stage (e.g., re-sampling, merging and splitting, compressing of recordings) is conducted using audio editing software ^4^. In general, recordings need to be visualized using spectrograms (i.e., sonograms) and played back to detect, identify, and then manually annotate the target sounds (usually sonant animal species) ^4, 5^. To our knowledge, no dedicated tool allows for a consistent, structured validation workflow of these manual annotations yet by independent experts. Increasingly, automated detection and classification of sounds is used to facilitate processing large amounts of audio data ^6^. The latter still need to be verified by human experts, who rely either on their own knowledge, or reference recordings found in audio repositories (i.e., reference sound libraries) linking recordings to species identities ^7^. Alternatively to their time-consuming manual annotation, soundscapes can be characterised with automatically computed eco-acoustic indices that can be linked to biodiversity metrics ^8, 9^, or with general acoustic feature sets that can be used to detect anomalous sound events in an unsupervised manner ^10^. Finally, in bioacoustics- or behavior-focused studies, but also for the identification of bats, the target sounds need to be analysed further by measuring their properties in the frequency-time-amplitude space ^11, 12^. At the time of writing, no software integrates all these different data processing stages into a consistent, integrated workflow, and reference libraries are still scarce for particular species groups ^1^.

Software tools that handle audio data need to be built sustainably to benefit a large user base in the research community. While the majority of software is free, few are online-based, many are specialised on specific taxa, and only half of them are open-source ( Table 1). It is essential to have free tools that all researchers and practitioners can use, irrespective of their budget constraints. Also, only open-source projects guarantee that they can be continuously developed to keep up with the pace of technological progress, that they stay accessible over time, and that the actual functions are transparent and replicable. Within two years, three out of the 19 reported software tools by Priyadarshani *et al.* ^6^ appear to have ceased development. Accessibility, which is essential for international collaboration and verification of bioacoustic data ^13^, also requires online solutions that are mostly independent of operating systems or any commercial software. In a nutshell, the field of ecoacoustics requires an open-source, online tool, as this fulfils most requirements: being free, easily maintainable, collaborative and accessible.

**Table 1.** Overview of currently available software tools for ecoacoustics. We included only ecoacoustics software tools built specifically for ecoacoustics that can play back audio and represent audio visually in the form of spectrograms. We excluded tools that were not developed in the last 2 years.

Tool	Soundscape recordings	max frequency (kHz)	manual annotation	automated species detection	Reference recordings	Taxa	Sound analysis	Acoustic indices	Access	License	Installation	Interaction
Animal Sound Identifier	no	unknown	yes	yes	yes	all	yes	no	free (but requires Matlab)	unknown	package	command line
Arbimon	yes	22.05	yes	yes	yes	all	yes	yes	free and commercial	proprietary	none	GUI
Avisoft‐SASLab Pro	yes	unknown	yes	yes	yes	all	yes	yes	commercial	proprietary	local	GUI
BatSound	yes	192	yes	no	no	bats	yes	no	commercial	proprietary	local	GUI
Biosounds	yes	192	yes	no	yes	all	partly	no	free	GPLv3	server	GUI
eBird	yes	unknown	no	yes	yes	birds	no	no	free	proprietary	server	GUI
Ecosounds	yes	unknown	yes	no	yes	all	partly	no	free	Apache 2.0	server	GUI
eXtensible BioAcoustic Tool (XBAT)	unknown	unknown	yes	yes	unknown	all	yes	unknown	free	GPL-2	package	command line
Ishmael	no	96	yes	yes	yes	all	yes	yes	free	unknown	local	GUI
Kaleidoscope Pro	yes	unknown	yes	yes	?	all	yes	yes	commercial	proprietary	local	GUI
Luscinia	yes	unknown	no	no	no	all	yes	yes	free	unknown	Java	GUI
monitoR	no	no limit	yes	yes	no	all	yes	no	free	GPL-2	package	command line
PAMGuard	no	24	yes	yes	no	marine	no	yes	free	GPL-2	local	GUI
Raven Pro	no	unknown	yes	yes	no	all	yes	yes	commercial	proprietary	local	GUI
seewave	no	unknown	yes	yes	no	all	yes	yes	free	GPL (>= 2)	package	command line
SIGNAL	no	unknown	yes	yes	no	all	yes	no	commercial	proprietary	local	GUI
Sonobat	yes	unknown	yes	yes	unknown	bats	yes	unknown	commercial	proprietary	local	GUI
Sound Analysis Pro	no	88.2	yes	yes	no	birds	yes	yes	free	GPL-2	local	GUI
SoundID	unknown	unknown	no	yes	yes	all	yes	yes	commercial	proprietary	local	GUI
Tadarida	no	250	no	yes	no	all	yes	yes	free	CC-BY, LGPL- 3.0, GPL-3.0	local	GUI
warblR	no	no limit	yes	yes	no	all	yes	unknown	free	GPL (>= 2)	package	command line
Xeno-Canto	yes	unknown	no	no	yes	birds	no	no	free	CC	none	GUI

We present BioSounds: an open-source, online platform for ecoacoustics, designed by ecologists and built by software engineers. Currently, BioSounds can be used to manage soundscape and reference recording collections, to manually create and independently review annotations in recordings, and to perform basic sound measurements in time and frequency. BioSounds was originally based on Pumilio ^3^ but the latter has ceased development and Biosounds has considerably expanded since. At the moment of writing, only one other software - Ecosounds - offers similar functions as BioSounds ^5^, and we compare them with each other ( Table 2). We detail the structure and functionality of BioSounds in the following and announce our development goals.

**Table 2.** Differences between BioSounds and Ecosounds. We compared both software tools’ functionalities that are relevant for administrators and normal users. Both tools work with Chrome, Firefox, and Internet Explorer browsers.

Category	Criteria	Biosounds	Ecosounds
Management	Accessibility	Open collections available. Users registered by administrators on demand. Users can access all collections.	Open collections available. Self-registration of users. Project owners can define user access.
	Organisation	Creation of collections by administrator via database. Option for collection description.	Creation of projects by user via website interface. Option for project image, description, and location (visible on map).
	Recordings	Administrators can upload, delete, rename sound recordings. Recording size limit: 300 MB.	No upload of audio files directly through website possible; manual inspection of data and quality control by main administrator. Recording limit: from 1 hour to 1 day (>1GB)
	Reference collection	Reference collections available with dedicated list view. Tags can be marked as reference.	Annotation library of animal sounds in extra sub-menu available (1390 recordings). Tags in spectrograms can be marked as reference.
	Long soundscapes	Spectrogram range limited by 300 MB file size limit.	Multiple recordings can be displayed on large temporal scales across project sites.
	Statistics	Overview of users accessible to administrator.	Statistics of annotations, projects, sites, recordings, users, etc. can be displayed.
	Playback duration	Logged for each user and recording, accessible to admin in database.	not available
	Guide	Online public user guide	not available
Annotation	Database	Species names of birds, bats and frogs, mainly of Southeast Asia, can be entered in Latin or English. Species list can be expanded by admins in database.	Species names and of birds, frogs, mammals and other sounds, mainly from Australia, can be entered in Latin or English.
	Creation	Creation of new annotation (tag) via spectrogram selection and button click.	Annotation automatically created via spectrogram selection.
	Editing	Editing, deleting of annotation in pop-up window. Option to estimate call distance. No option to copy or move tags.	Editing and deleting of annotation in main window. No option to copy or move tags.
	Review	Dedicated function for species validation by users with reviewing privileges. Annotations can be zoomed.	not available
	Identification	Direct link to Google images and Xeno- Canto to check species identification.	not available
	Download	Download by administrator through database.	Download in CSV by user through website interface.
Recording	Playback	Play/pause, stop button below spectrogram. Cursor can be dragged. Option for continuous playback.	Play/pause button below spectrogram. Cursor can be dragged. Option to rewind, fast forward and rewind recording (30 s).
	Analysis	Time and frequency coordinates displayed for current spectrogram or selection, can be exported to clipboard via button.	Time displayed below spectrogram. Frequency only displayed for selections.
	Ultrasound	Up to 192 kHz in Chrome and Firefox. Playback speed can be adjusted between 0.05 and 1 x.	not available
	Filtering	Filtering of sound frequencies outside zoomed selection, checked by default (what you see is what you hear).	not available
	Navigation	Any part of spectrogram in time and frequency can be navigated to and zoomed into.	Only navigation along time axis.
	Visibility	Left and right audio channels can be displayed separately for stereo recordings. FFT window size set by administrator through website interface.	No option to change audio channel or FFT window size.
	Download	Recording: MP3 or OGG (ultrasound). Spectrogram: PNG	Recording: WAV or MP3. Spectrogram: PNG

## Methods

### Implementation

**Figure 1.**
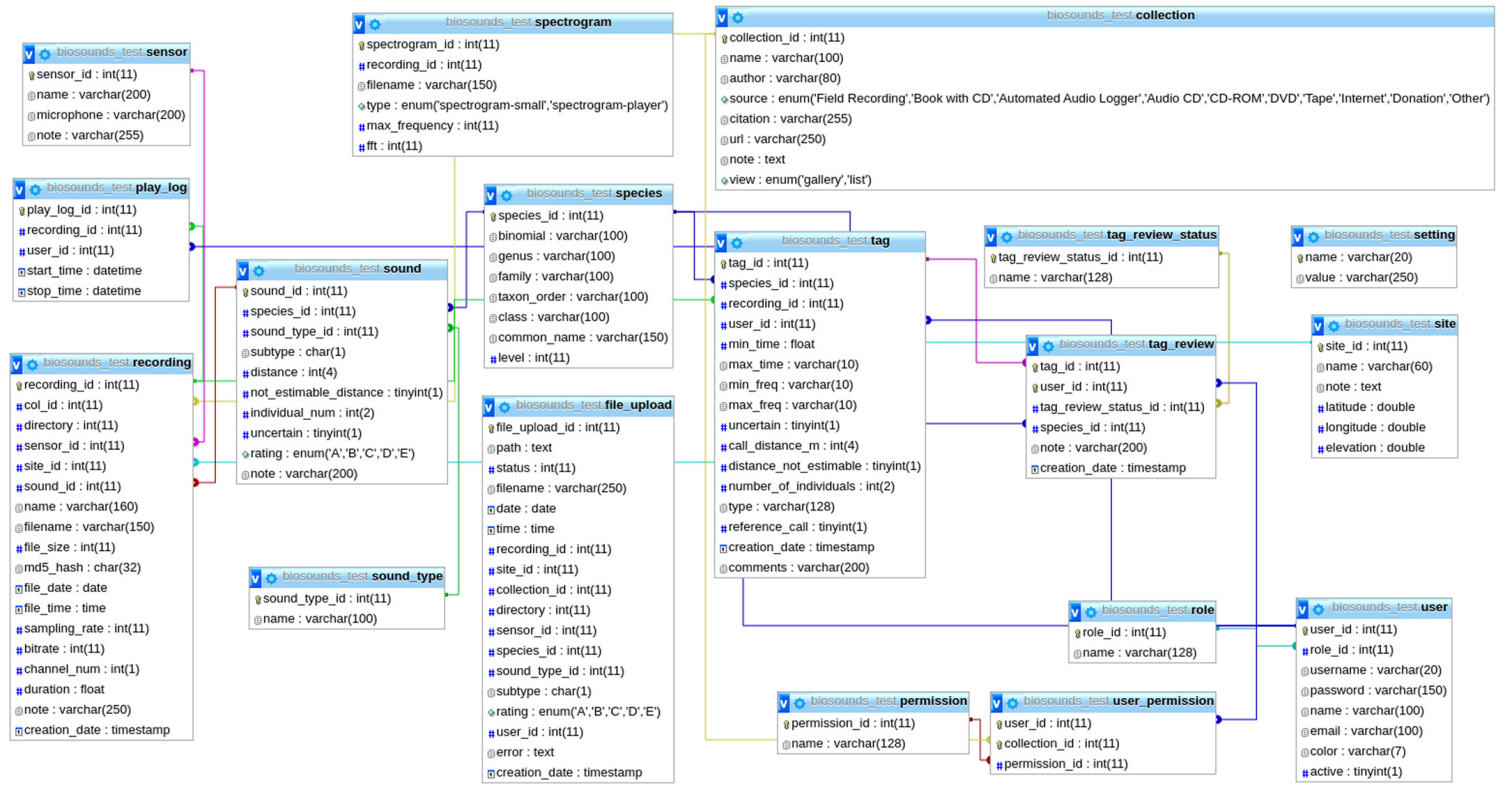
MySQL database structure in BioSounds.

***Coding languages, libraries, and tools*.** BioSounds is a web-based application written in PHP 7 ^14^, Python 2.7 ^15^, Javascript ^16^, JQuery 3.4 ^17^, Twig 2 ^18^, CSS ^19^ and HTML 5 ^20^. It uses Web Audio API ^21^, Sox 14.4 ^22^, Lame ^23^ and ImageMagick ^24^ software for sound and image processing, a MySQL ^25^ database for organising the data ( Figure 1), a RabbitMQ ^26^ queue for file processing, Plupload 1.5 as a visual file upload tool ^27^, JQuery UI 1.12 ^28^, JCrop 0.9 ^29^, Bootstrap 4.3 ^30^ and the Symfony 4 process component ^31^ for managing the scripts execution. The Python libraries used are: Numpy ^32^, Pillow ^33^ and Audiolab 0.8 ^34^. We containerized the project using Docker ^35^, which spares software developers the time for installing libraries, the database, and configuring the server. This setup allows developers to run the project on their machines quickly and free of typical installation issues like library version incompatibilities.

***Audio visualization and playback*.** The core sound visualisation and playback tasks are handled by two distinct components. First, spectrogram images are generated by the Python script ‘sound visualization tool’, which was created for the discontinued ‘Pumilio’ project ^3^. This script generates spectrograms by computing a Fast-Fourier Transform on the waveform of the audio recording. Second, sound playback and speed control use Web Audio API, a high-level application programming interface for processing and synthesizing audio in web applications. It is included in modern browsers to take advantage of the browser resources without requiring any extra media player or library in our project, and we plan to use it for generating spectrograms too.

### Operation

***Server installation*.** BioSounds is published in a GitHub repository ^36^ and needs to be installed in a web server to run. Instructions and general information regarding the setup for developers and the production server are included in the README file on GitHub. The BioSounds installation for local development (in the developer’s machine) is facilitated by a Docker setup. We provide a set of Docker configuration files that can also aid the server installation, but the final setup should be carried out by the server administrator (or devOps engineer) of the institution. For server installations without Docker, a step-by-step installation guide is provided in the repository.

***Access*.** We run an online instance of BioSounds for our project SoundEFForTS ^37^, where most of the steps described in the use cases below can be reproduced. This website can host public reference collections (i.e., reference audio libraries) for prospective users, for instance for Chiroptera and Anura. Soundscape collections, due to their larger size, can be integrated up to a manageable size for projects contributing to BioSounds development.

Users can access BioSounds (both the existing instance and future installations) via a desktop browser with an internet connection. BioSounds works with Windows, Linux, and MacOS operating systems and the most common internet browsers (Firefox, Chrome, Safari). 

***Collections*.** BioSounds organises audio recordings (named “recordings” hereafter) within collections. Collections can be accessed through the "Collections" drop-down menu. Those that are part of ongoing research projects are only visible to registered users; open collections are public. Collection creation is still handled directly via the database by adding it to the table ´Collection´. Administrators can then upload recordings in most common audio formats into collections. PNG image previews of the spectrograms and MP3s of the audio file are generated after insertion into the database. Audio recordings can be given names that differ from the default file name. Collections can be shown with a gallery view (thumbnails with sound names) or a list view with larger spectrograms and a simple audio player, and comments can be inserted. There are two types of collections in BioSounds: soundscape recording collections ("soundscape collections" hereafter) and reference recording collections (i.e., reference audio libraries; named "reference collections" hereafter).

**Figure 2.**
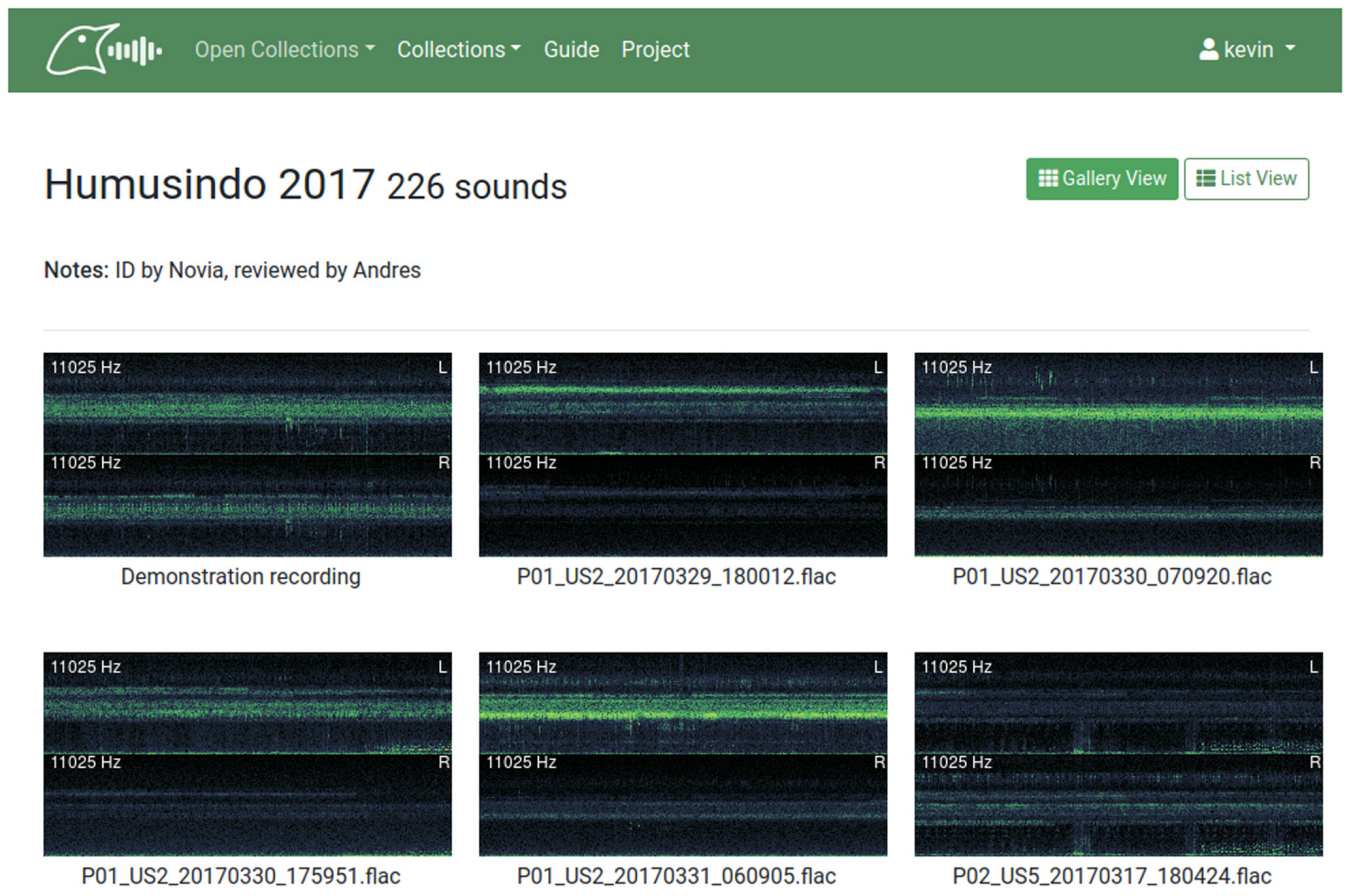
The default gallery view for soundscape recording collections in BioSounds.

Soundscape collections contain field recordings which each encompass a range of sounds from a particular site during a particular time interval. The recordings within are displayed with the gallery view by default, which shows either mono or stereo thumbnails of their spectrograms along with the sound names and the overlaid maximum recorded sound frequency ( Figure 2).

**Figure 3.**
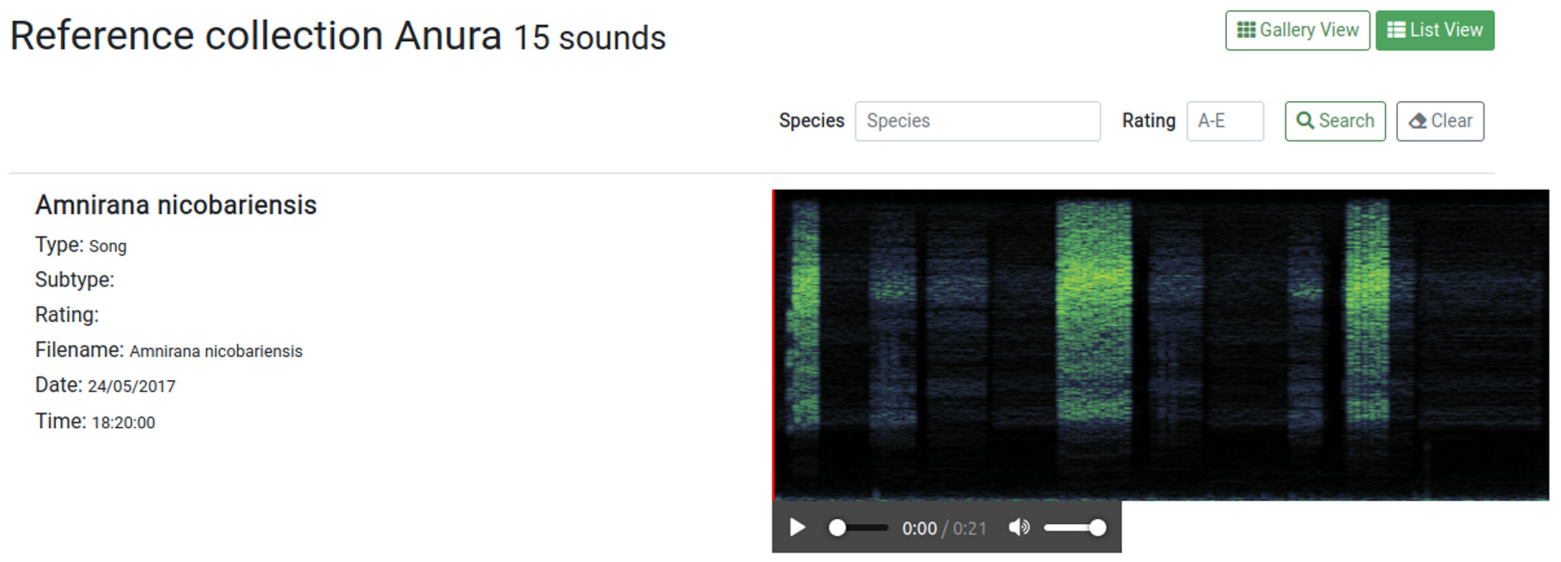
Reference recording shown in list view. Reference collections can be filtered by species and rating, and additional information regarding the recording is displayed. Reference recordings can be played back directly in list view.

Reference recording collections link individual recordings to identified sound sources (typically, sonant animal species). They display recordings with a list view by default; an example is shown in the public “Reference collection Anura”. Reference collections can host recordings that are needed for supporting the identification of the animals of particular taxa or regions. The spectrogram in the list view gives a rapid overview of the reference sound, and the embedded audio player can play it back directly ( Figure 3). In contrast to soundscape recordings, uploaded reference recordings need to be assigned to animal species, and they can have a vocalisation type and quality rating. Reference collections can be filtered by species and rating.

***Users*.** BioSounds has two registered user classes: normal users and administrators. All registered users can open all recordings inside the spectrogram player, as well as create, view, and edit their own annotations (called "tags" in BioSounds) that are linked to sound sources (mostly sonant animal species). Normal users have viewing and reviewing privileges for other users' tags that are set by administrators for single collections. Administrators can view, review, and edit all users' tags. They can also create users, set their tags' color, and define their status (normal user/administrator). Finally, administrators can upload, rename, and delete recordings.

**Figure 4.**
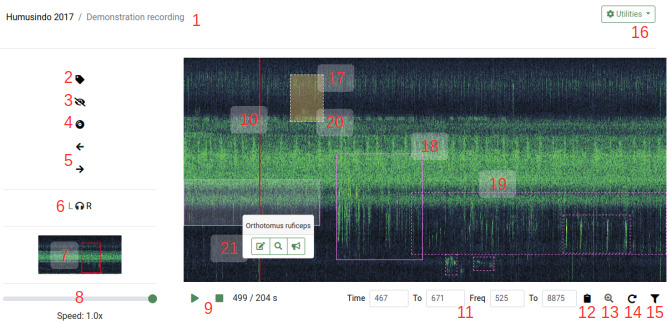
BioSounds spectrogram player. 1: sound and collection name. 2: annotating/tagging sounds. 3: hiding/showing tags. 4: playback mode. 5: moving window left and right. 6: audio channel selection. 7: overview spectrogram, red rectangle shows current view. 8: playback speed. 9: playback/pause and stop, time position. 10: playback cursor. 11: time (s) and frequency (Hz) coordinates of current view or selection. 12: copying time and frequency coordinates. 13: zooming. 14: continuous playback. 15: frequency filter. 16: utilities: image and audio download, file info. 17: tags of different users shown with different colors. 18: reviewed tags with solid border. 19: not yet reviewed tags with dashed border. 20: tags without detection distance with orange shading. 21: tag species appears on click, with buttons for editing, zooming, and estimating distance.

***Spectrogram player*.** Recordings can be opened in the spectrogram player ( Figure 4). Spectrograms are visualisations of sound where sound amplitude is shown in color or grayscale tones, time is shown on the X axis, and frequency is displayed on the Y axis. The spectrogram player offers various functionalities for tagging sounds: it is possible to play back sound, filter frequencies, navigate the spectrogram, assign selections to animal species (or other sound sources), and perform basic sound analysis.

## Use cases

### Bird community analysis

**Figure 5.**
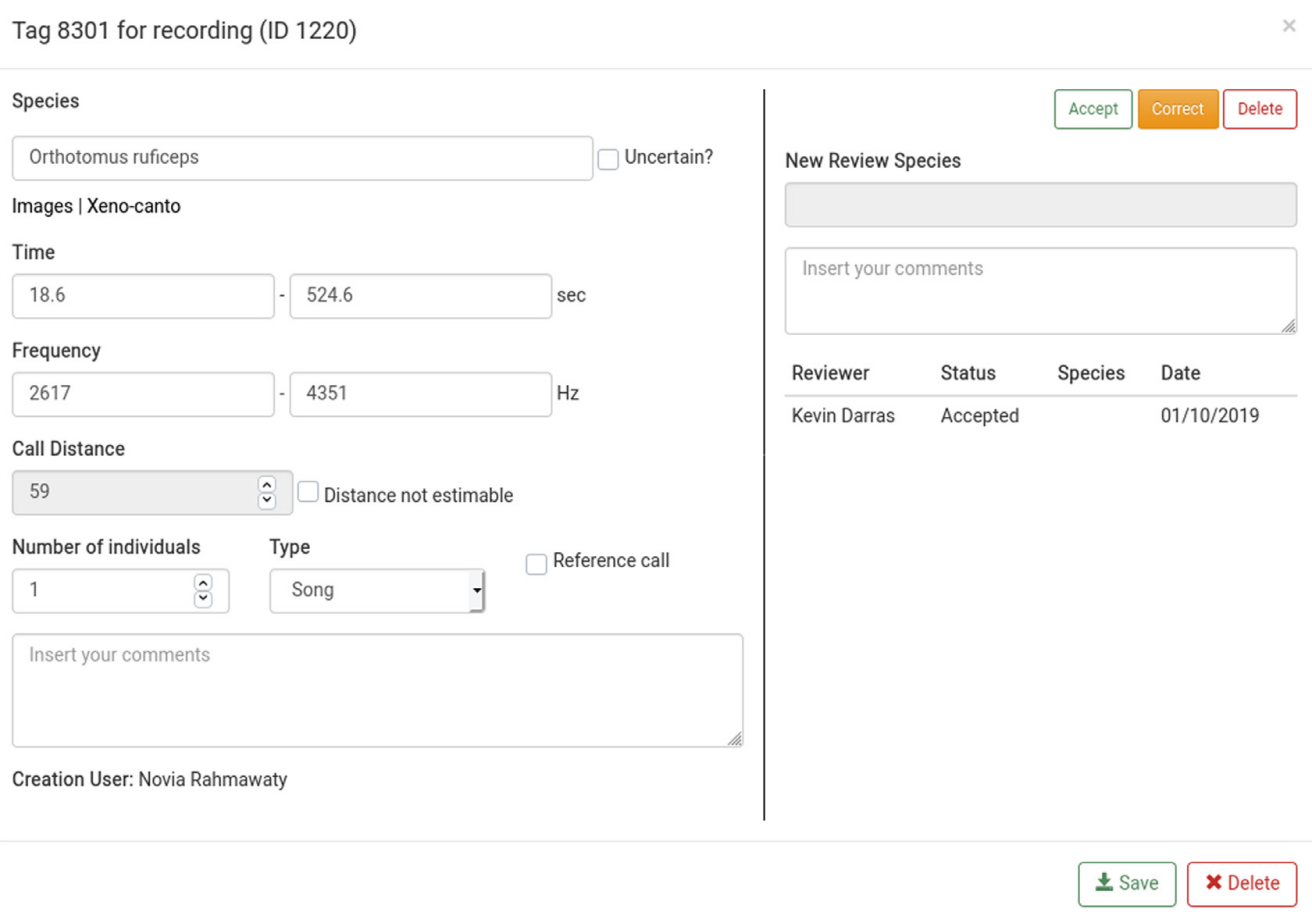
The tag editing window. The right pane is only visible to users with reviewing privileges.

Soundscape recordings can be annotated manually and reviewed by expert ornithologists, as exemplified in the collection " Upland plots dry season 2013". Users can scan recordings visually and aurally using the built-in reading mode, which zooms to a 60 s long section of the recording, including all frequencies, and enables continuous playback. All avian species can be tagged/annotated based on rectangular spectrogram selections along the frequency and time axes. Species are chosen from the integrated species list, and links to Xeno-canto and Google image searches direct the user to the selected species to support identification ( Figure 5). Project-specific reference collections can also be consulted to confirm species identification. Unclear identifications can be marked as uncertain. Coordinates (in time and frequency) are saved automatically based on the boundaries of the selection. Tags can be designated as reference recordings for future inclusion into reference recording collections; comments can be inserted. Tags can be zoomed into and any of the current (filtered or unfiltered) spectrogram views (image or audio) can be downloaded for sharing with collaborators. Distances are estimated in a standardised way using a function that enables full-spectrum viewing and playback of the tags based on a spectrogram of the first 30 s of the tag. Reference audio recordings of test tones emitted at known distances are needed (see recording "Sound transmission - full spectrum" in Demo collection) to estimate detection distances in an unbiased way ^38^.

An advantage of automated acoustic survey data is that they can be validated on multiple levels, yielding accurate datasets ^39^. In BioSounds, it is possible to review tags for validating species identification as well as auxiliary tag data. Administrators can grant tag reviewing privileges to users other than the creator. Users with reviewing privileges can either accept species identifications, revise them by suggesting other species, or reject them by marking the annotation for deletion ( Figure 5). Administrators can also check the listening durations of each user for each recording to verify that all recordings have been listened to in entirety, and to extract a measure of the sampling intensity. Finally, it is possible to train users by letting them learn from other users’ annotations after granting them viewing privileges, and thereafter, to test their performance with already annotated recordings where the annotations are invisible to the user being tested. After the validity checks have been run, administrators can export the tag data through a MySQL database administration tool like phpMyAdmin ^40^ for further statistical analysis.

### Bat community analysis

Soundscape recordings that span the ultrasound frequency range (i.e., ultrasoundscapes) can be similarly analysed with the same functions as for the bird community analysis use case, but they present specific challenges regarding the analysis of bat calls. Most importantly, ultrasound is not audible, so that users need to use the playback speed slider to reduce the playback frequency to artificially hear the ultrasound calls. This can be tested with the annotated example recording "Sample Ultrasoundscape" that is uploaded in the " Demo" collection; any playback rate from 0.05 to 1 can be chosen. To aid in bat call visualisation, the spectrogram settings can also be adjusted by administrators to choose different FFT window sizes.

However, bat species identification is more challenging as bat calls from different species can be similar. Thus, we included bat morphospecies (to be exact, morphocall types) named with single letters from A to J into the species list, suffixed with digits to designate different call types from the same species. Exact measurement of bat call features (such as start and end frequency, frequency of maximal energy, as well as call and call interval duration) usually determines the assignment bat calls to specific species: using the clipboard button ( Figure 4), users can copy the frequency and time coordinates of the current selection to the clipboard to perform basic sound analysis. The exported values can be readily pasted into spreadsheets, and bat call metrics of interest can be rapidly computed with formulae. For those species that have taxonomically unequivocal calls, the users can refer to the reference collection to corroborate their identifications. Finally, manual distance estimation of bat calls is impractical due to their mobility and the fact that we cannot intuitively estimate the distances of human-inaudible sounds, so that the tags can be marked as having not estimable distances.

## Conclusions

BioSounds can be used to archive, visualise, play back, and share soundscape recordings online with users that have different access privileges. The recordings can be analysed collaboratively for detecting sonant animal species such as birds and bats as to derive measures of their activity for use in ecological studies. BioSounds has already been used successfully to analyse bird communities ^41^ and to measure bat activities ^42^, while performing basic sound analysis. Region- and taxon-specific reference collections can be created, like the anuran calls collection we currently host ^43^.

We strive to expand the functionality of BioSounds and keep it accessible in the long term. Open access is a requirement for future development and maintenance. However, it is not a guarantee for a sustainable project either, as some of the open-source projects listed by Priyadarshani *et al.* in 2018 ^6^ are currently discontinued projects. In Biosounds, we refactored the original Pumilio project, implemented best coding practices, and used development tools, like Docker, all of which facilitate developers’ work and help them engage in collaboration. We welcome new collaborators to support the project development who could become co-authors on subsequent versions of this article. Among others, we plan to develop the following functions:

- Automated detection and classification of vocalisations, using existing tools ^44, 45^
- Computation of acoustic diversity indices to monitor biodiversity ^46^
- Developing the sound analysis tool to include the sound amplitude dimension
- Linking BioSounds to taxonomic databases for an exhaustive, up-to-date list of sonant animals
- Managing and displaying geographic locations of recordings ^3^
- Displaying multiple recordings of single sites on a common time axis to visualise longer soundscapes ^5^

## Data availability

All the recordings referred to here are accessible in open collections without login on our online instance of BioSounds: https://soundefforts.uni-goettingen.de/biosounds/.

## Software availability

Source code available from: https://github.com/nperezg/biosounds

**Archived source code at the time of publication:** http://doi.org/10.5281/zenodo.4047711 ^47^.

**License:** GNU General Public License v3.0 (GPLv3).
