# ecoSound-web: an open-source, online platform for ecoacoustics

**DOI:** 10.12688/f1000research.26369.2

**Published:** 2023-03-01

**Authors:** Kevin F.A. Darras, Noemí Pérez, Mauladi -, Liu Dilong, Tara Hanf-Dressler, Matthias Markolf, Thomas C Wanger

**Affiliations:** 1 Computational Landscape Ecology, TU Dresden, Dresden, Sachsen, 01737, Germany; 2 Agroecology, University of Göttingen, Göttingen, Niedersachsen, 37077, Germany; 3 Sustainable Agricultural Systems & Engineering Laboratory, School of Engineering, Westlake University, Hangzhou, 310030, China; 4 Department of Information Systems, Universitas Jambi, Jambi, Jambi, 36122, Indonesia; 5 Quality Technology Centre, Nanjing Julong Steel Pipe Co., Ltd., Nanjing, 211800,, China; 6 Behavioral Ecology & Sociobiology Unit, German Primate Centre,, Göttingen, Niedersachsen, 37077, Germany; 7 Key Laboratory of Coastal Environment and Resources of Zhejiang Province, Westlake University, Hangzhou, China

**Keywords:** Soundscape, sound analysis, ecoacoustics, passive acoustic monitoring, automated sound recording, autonomous recording units, spectrogram, audio annotation

## Abstract

Passive acoustic monitoring of soundscapes and biodiversity produces vast amounts of audio recordings, but the management and analysis of these raw data present technical challenges. A multitude of software solutions exist, but none can fulfil all purposes required for the management, processing, navigation, analysis, and dissemination of acoustic data. The field of ecoacoustics needs a software tool that is free, evolving, and accessible. We take a step in that direction and present ecoSound-web: an open-source, online platform for ecoacoustics designed and built by ecologists and software engineers. ecoSound-web can be used for storing, organising, and sharing soundscape projects, manually creating and peer-reviewing annotations of soniferous animals and phonies, analysing audio in time and frequency, computing alpha acoustic indices, and providing reference sound libraries for different taxa. We present ecoSound-web’s features, structure, and compare it with similar software. We describe its operation mode and the workflow for typical use cases such as the sampling of bird and bat communities, the use of a primate call library, and the analysis of phonies and acoustic indices. ecoSound-web is available from: https://github.com/ecomontec/ecoSound-web

## Introduction

Automated passive acoustic recording methods are powerful means for monitoring biodiversity and the environment in ecological research, i.e., the field of ecoacoustics. The resulting soundscape recordings - comprising all sounds recorded in a sea- or landscape ^1^ - present new opportunities for ecologists. However, they yield huge amounts of data that are challenging to manage ^2^ and to analyse for extracting their ecological information – such as biodiversity, human activities, or geophysical events. Overall, soundscape ecologists require a dedicated tool that allows for such a comprehensive workflow ^3^, and which aligns with FAIR research principles ^4^.

Methods for the annotation and analysis of audio recordings still undergo rapid development. Annotations are increasingly generated with automated methods ^5^ to forego laborious but common manual annotation by humans. However, even reference machine learning tools such as BirdNET ^6^ require post-processing to yield usable results. More importantly, sound source identifications from humans and machines need to be cross-checked, by peers or experts, who rely on reference recordings found in sound libraries to ascertain the identification of sound sources ^7^. Alternatively or in addition to taxonomic annotation of recordings, soundscapes can be characterised with automatically-computed acoustic indices that can measure spectral and temporal variation, entropy, or complexity, and be linked to biodiversity metrics ^8– 11^. General acoustic feature sets can also be used to detect anomalous sound events in an unsupervised manner ^12^. In marine ecoacoustics, annotating and quantifying the temporal proportion of phonies (i.e., sounds of biological, geophysical, and human origin) is well-established ^13^, and the sound pressure levels from calibrated equipment are a common metric for studying noise impacts on biological activity ^14, 15^. Finally, in bioacoustics- or ethological studies, but also for the identification of bats ^16^ and soundscape characterisation ^17^, the target sounds need to be analysed further by measuring their properties in the frequency-time-amplitude space ^18, 19^. At the time of writing, no software integrates all these different data processing stages into a consistent, integrated workflow for ecoacoustic projects across realms, taxa and regions. Reference sound or call libraries are also still scarce for particular species groups ^2, 20^, even though recent advances were made for Orthopterans on Xeno-Canto ^7^ (additionally to well-studied birds), and bats on ChiroVox ^21^.

Software tools that handle audio data need to be built sustainably to benefit a large user base in the research community and to stimulate research ^22^. While the majority of tools are free, few are online-based, many are specialised on specific taxa, realms or regions, only some are open-source, and most cover only parts of the workflow described earlier. It is essential to have free tools that all researchers and practitioners can use, irrespective of their budget constraints. Also, only open-source projects, in conjunction with long-term vision and funding ^22^, guarantee that they can be continuously developed to keep up with the pace of technological progress, that they stay accessible over time, and that the actual functions are transparent and replicable. Accessibility, which is essential for international collaboration and verification of ecoacoustic data ^23^, also requires online solutions that are mostly independent of operating systems or commercial software. Finally, tools that integrate multiple steps of the workflow outlined earlier will be inherently less complex for users, more practical, and more replicable than separate, specialised solutions. In a nutshell, the field of ecoacoustics requires an open-source, online, comprehensive software tool.

Here, we first provide an up-to-date overview of software tools available for ecoacoustics. We then introduce ecoSound-web: an open-source online platform for ecoacoustics, designed and built by ecologists and software engineers (for the related GitHub project see: https://github.com/ecomontec/ecoSound-web). Currently, ecoSound-web can be used to 1) upload and organize soundscape recordings within collections; 2) to manage users and access to collections within dedicated projects; 3) visualize them on maps and timelines; 4) play back, navigate, and filter their sound and spectrograms; 5) create and peer-review manual recording annotations; and 6) measure sounds and compute acoustic indices. ecoSound-web was forked from BioSounds (c.f. article version 1). We detail the structure and functionality of ecoSound-web in the following and announce our development goals.

## Methods

### Implementation

**Figure 1.**
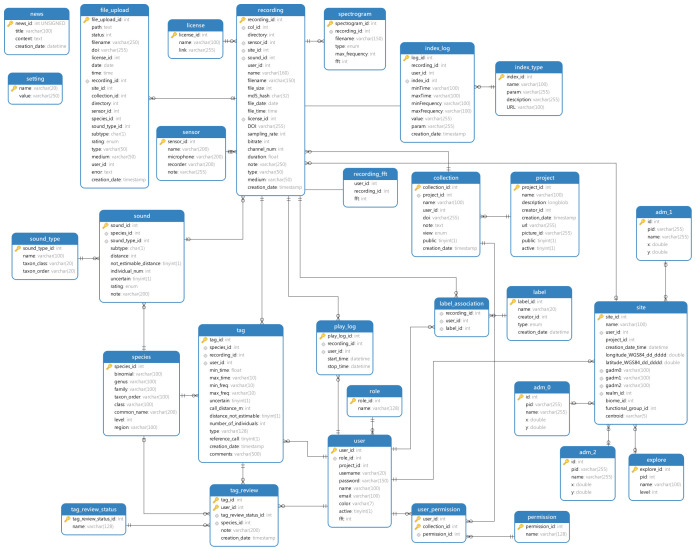
MySQL database structure in ecoSound-web.

***Coding languages, libraries, and tools.*** ecoSound-web is a web-based application written in PHP 7 ^24^, Python 2.7 and 3.10 ^25^, Javascript ^26^, JQuery 3.4 ^27^, Twig 2 ^28^, CSS ^29^ and HTML 5 ^30^. It uses Web Audio API ^31^, Sox 14.4 ^32^, Lame ^33^, ImageMagick ^34^ and Scikit-maad 1.3.12 ^35^ software for sound and image processing, a MySQL ^36^ database for organising the data ( Figure 1), a RabbitMQ ^37^ queue for file processing, Plupload 1.5 as a visual file upload tool ^38^, GADM as administrative regions for the sites ^39^, JQuery UI 1.12 ^40^, JCrop 0.9 ^41^, Bootstrap 4.3 ^42^, Leaflet ^43^, Timeline.js ^44^, Bootstrap-selected ^45^, Jquery.cookie ^46^, DataTables ^47^ and the Symfony 4 process component ^48^ for managing the scripts execution. Further Python libraries used are: Numpy ^49^, Pillow ^50^, Audiolab 0.8 ^51^, Matplotlib ^52^, SciPy ^53^ and Scikit-image ^54^. We containerized the project using Docker ^55^, which spares software developers the time for installing libraries, the database, and configuring the server. This setup allows developers to run the project on their machines quickly and free of typical installation issues like library version incompatibilities.

***Audio visualization and playback.*** The core sound visualisation and playback tasks are handled by two distinct components. First, spectrogram images are generated by the Python script ‘sound visualization tool’, which was created for the discontinued ‘Pumilio’ project ^56^. This script generates spectrograms by computing a Fast Fourier Transform on the waveform of the audio recording, at a user-specified window size (at 128, 256, 512, 1024, 2048, or 4096). Second, sound playback and speed control use Web Audio API, a high-level application programming interface for processing and synthesizing audio in web applications. It is included in modern browsers to take advantage of the browser resources without requiring any extra media player or library in our project.

### Operation

***Ecoacoustic workflow.*** In ecoacoustics, a general workflow is currently not comprehensively defined. We therefore combine insights from different literature sources and our own experience to propose a general workflow as follows ( Table 1): 1) Data management: Acoustic data need to be backed up, archived, and organized according to space, time, and other meta-data ^56^. 2) Signal processing: Recordings can be amplified, re-sampled, split, filtered, compressed, etc. for facilitating the workflow ^57^. 3) Audio navigation: Sound recordings can be visualized with spectrograms (i.e., sonograms) or waveforms, and played back. 4) Recording annotation: specific spectro-temporal regions can be interpreted and annotated with the sound source identity or comments. 5) Acoustic analysis: Spectral, temporal, and amplitudinal properties of the recordings can be measured or summarised with acoustic indices ^10^. 6) Collaboration: The raw and secondary data can be shared with collaborators or the public. In the following we introduce ecoSound-web to enable the ecoacoustic community to follow and use parts of the acoustic workflow introduced here.

**Table 1.** Overview of currently available software tools for ecoacoustics. We included only ecoacoustics software tools built specifically for ecoacoustics that fulfilled at least one of the overarching purposes of the ecoacoustic workflow. We excluded tools that were not developed in the last 2 years.

Software tools	URL	Scope			1. Data management					2. Signal processing				3. Audio navigation					4. Recording annotation			5. Acoustic analysis					6. Dissemination					Product information								
		Taxonomic	Regional	Ecological	Backup and retrieval	Indexing/ organizing/ labeling	Archival/ long- term storage (>10 years)	Geographic management	Temporal management	Amplification	Snippet extraction	Noise reduction/ addition	Resampling	Spectograms	Waveforms	Frequency filtering	Playback	Playback rate/ pitch control	Manual annotation	Automated sound detection	Reference recordings	Acoustic indices	Frequency spectrum	Frequency- time measurements	Sound level measurements	SPL calibration	Public projects	Collaborative projects	Discussion platform/ fora	Form to contact contributors	Research indices	User access	Code access	Link to code	License	Software type	Interaction	Execution	Last update	Manual
Animal Sound Identifier	https://onlinelibrary.wiley. com/doi/full/10.1111/ ele.13092	all	all	all	local	no	no	no	no	unknown	yes	yes	yes	yes	no	yes	yes	no	yes	yes	no	no	unknown	unknown	unknown	no	no	no	no	no	no	free (but requires Matlab)	open		unknown	package	command line	Matlab	2019	https://onlinelibrary.wiley.com/action/ downloadSupplement?doi=10.111 1%2Fele.13092&file=ele13092-sup- 0001-SupInfo.pdf
Arbimon	https://arbimon.rfcx.org/	all	all	all	yes	yes	unknown	yes	yes	yes	yes	yes	unknown	yes	no	yes	yes	unknown	yes	yes	no	unknown	unknown	unknown	unknown	unknown	yes	yes	no	yes	no	free and commercial	closed		proprietary	online	graphical user interface	web browser	2023	https://b8bb32e8-00e2-4e12-8f8f- 84de462a5e7d.filesusr.com/ugd/13049a_ 2ef3587909004f99998ae77af49fe33c.pdf
Avisoft‐SASLab Pro	http://www.avisoft. com/sound-analysis/	birds, mammals, rodents, frogs, fish, insects	all	all	local	yes	no	yes	yes	yes	yes	yes	yes	yes	yes	yes	yes	yes	yes	yes	no	yes	yes	yes	yes	yes	no	no	no	no	no	commercial	closed		proprietary	local	graphical user interface	Windows	unknown	http://www.avisoft.com/SASLabPro.pdf
BatSound	https://batsound.com/	bats	all	terrestrial	yes	yes	no	no	yes	yes	yes	yes	no	yes	yes	yes	yes	yes	yes	no	no	no	yes	yes	yes	yes	no	yes	no	no	no	commercial	closed		proprietary	local	graphical user interface	Windows	unknown	https://batsound.com/inc/files/pdf/ BatSoundManual44.pdf
**ecoSound-web**	https://ecosound-web.de/	fauna	all	all	yes	yes	unknown	yes	yes	yes	yes	no	no	yes	no	yes	yes	yes	yes	no	yes	yes	yes	yes	yes	no	yes	yes	no	no	no	free	open		GPLv3	online	graphical user interface	web browser	2023	not available
Ecosounds	https://www.ecosounds.org/	fauna	all	terrestrial	yes	yes	unknown	yes	yes	yes	yes	no	no	yes	no	unknown	yes	no	yes	yes	no	yes	yes	unknown	unknown	unknown	yes	yes	no	yes	yes	free	open		Apache 2.0	online	graphical user interface	web browser	2023	http://eprints.qut.edu. au/79388/5/79388.pdf
Ishmael	https://bioacoustics. us/ishmael.html	cetaceans	all	aquatic	no	yes	no	no	no	yes	no	yes	yes	yes	yes	yes	yes	yes	yes	yes	no	unknown	yes	yes	yes	unknown	no	no	no	no	no	free	open		unknown	local	graphical user interface	Windows	2019	https://bioacoustics.us/docs/ Ishmael_3.0_User_Guide.pdf
Kaleidoscope Pro	https://www.wildlifeacoustics. com/products/ kaleidoscope-pro	fauna	all	all	yes	yes	yes	yes	yes	yes	yes	no	unknown	yes	yes	yes	yes	yes	yes	yes	yes	yes	yes	yes	yes	yes	no	yes	no	no	no	commercial	closed		proprietary	local	graphical user interface	Windows, Linux, MacOS		https://www.wildlifeacoustics. com/uploads/user-guides/Kaleidoscope- User-Guide.pdf
Luscinia	http://rflachlan.github. io/Luscinia/	fauna	all	all	yes	yes	yes	yes	yes	yes	yes	yes	no	yes	yes	yes	yes	yes	yes	yes	no	yes	unknown	unknown	no	no	no	yes	no	no	no	free	open		unknown	local	graphical user interface	Windows, Linux, MacOS		https://github.com/rflachlan/Luscinia/wiki
monitoR	https://cran.r-project. org/web/packages/monitoR/ index.html	birds	USA	all	no	no	no	no	no	no	yes	no	no	yes	no	yes	unknown	no	yes	yes	yes	no	no	no	unknown	no	no	no	no	no	no	free	open		GPL-2	package	command line	R		https://cran.r-project.org/web/ packages/monitoR/vignettes/monitoR_ QuickStart.pdf
PAMGuard	https://www.pamguard. org/download.php?id=105	cetaceans	all	aquatic	no	yes	no	yes	unknown	yes	yes	yes	no	yes	yes	yes	yes	no	yes	yes	yes	no	no	no	no	no	no	no	yes	no	no	free	open		GPL-2	local	graphical user interface	Windows, Linux, MacOS		https://www.pamguard.org/cms/ PAMGUARD%20Training%20Tutorial%20v 1_4_03%20Sept2017.pdf
Raven Pro	http://ravensoundsoftware. com/software/raven-pro/	fauna	all	all	no	no	no	no	no	yes	yes	yes	yes	yes	yes	yes	yes	yes	yes	yes	no	yes	no	yes	yes	no	no	no	no	no	no	commercial	closed		proprietary	local	graphical user interface	Windows, Linux, MacOS		http://ravensoundsoftware. com/wp-content/uploads/2017/11/ Raven14UsersManual.pdf
SeeWave	http://rug.mnhn.fr/seewave/	all	all	all	no	no	no	no	no	yes	yes	yes	yes	yes	yes	yes	unknown	no	no	no	no	yes	yes	yes	yes	yes	no	no	yes	no	no	free	open	https://github. com/cran/ seewave	GPL (>= 2)	package	command line	R	2021	http://cran.at.r-project.org/web/ packages/seewave/seewave.pdf
SIGNAL 5.06.12	http://www.engdes. com/sigwin/products/sigwin/ sig5.html	all	all	all	no	no	no	no	no	yes	yes	yes	yes	yes	yes	yes	yes	yes	yes	yes	no	yes	yes	yes	yes	no	no	no	no	no	no	commercial	closed		proprietary	local	graphical user interface	Windows	2022	not available
Sonobat	https://sonobat.com/	bats	North America, UK	terrestrial	no	yes	no	no	no	yes	yes	yes	yes	yes	yes	yes	yes	yes	yes	yes	yes	yes	yes	yes	yes	no	no	no	no	no	no	commercial	closed		proprietary	local	graphical user interface	Windows, MacOS		https://sonobat.com/ sonobat_basic_operations/assets/player/ KeynoteDHTMLPlayer.html#0
Sound Analysis Pro 2011	http://www.soundanalysispro. com/	fauna	all	all	no	yes	no	no	no	yes	yes	yes	yes	yes	yes	yes	yes	yes	yes	yes	no	yes	yes	yes	yes	no	no	no	no	no	no	free	open		GPL-2	local	graphical user interface	Windows		http://www.soundanalysispro.com/ manual-1/manual-pdf/at_download/file
Tadarida	https://github.com/YvesBas/	bats; bush- crickets	France	terrestrial	no	yes	no	yes	yes	no	yes	yes	no	yes	no	yes	no	no	yes	yes	yes	yes	no	yes	yes	no	unknown	unknown	no	unknown	no	free	open		CC-BY, LGPL-3.0, GPL-3.0	package	graphical user interface	R	2023	https://github.com/YvesBas/Tadarida-L/ blob/master/Manual_Tadarida-L.odt
warbleR	https://cran.r-project. org/web/packages/warbleR/ warbleR.pdf	birds	all	all	no	yes	no	no	no	yes	yes	yes	yes	yes	no	yes	unknown	no	unknown	yes	yes	yes	yes	yes	yes	yes	no	no	no	no	no	free	open		GPL (>= 2)	package	command line	R	2022	https://cran.r-project.org/web/packages/ warbleR/warbleR.pdf
XenoCanto	https://www.xeno-canto.org/	birds, grasshoppers	all	all	yes	yes	yes	yes	yes	no	no	no	no	yes	no	no	yes	no	no	no	yes	no	no	no	no	no	yes	yes	yes	unknown	unknown	free	closed		CC	online	graphical user interface	web browser		not available
OPUS	https://opus.aq/	cetaceans	all	aquatic	yes	yes	unknown	yes	yes	yes	unknown	unknown	unknown	yes	no	unknown	yes	yes	unknown	unknown	yes	unknown	unknown	unknown	unknown	unknown	yes	yes	unknown	unknown	unknown	free on request	closed		proprietary	online	graphical user interface	web browser		https://opus.aq/content.html#tutorial
SoundClass	https://besjournals. onlinelibrary.wiley. com/doi/full/10.1111/2041- 210X.13964	all	all	all	no	no	no	no	no	no	yes	yes	no	yes	no	yes	unknown	no	yes	yes	no	no	no	no	no	no	no	no	no	no	no	free	open		GPL-3	package	command line	R		https://cran.r-project.org/web/packages/ soundClass/soundClass.pdf
ohun	https://www.biorxiv. org/content/10.1101/ 2022.12.13.520253v1. abstract?%3Fcollection=	all	all	all	no	no	no	no	no	no	yes	no	no	yes	no	yes	yes	no	no	yes	no	no	no	no	no	no	no	no	no	no	no	free	open		NA	package	command line	R		https://cran.r-project.org/web/packages/ ohun/vignettes/ohun.html
Wildtrax	https://www.wildtrax. ca/home.html	predominantly birds	Canada	all	yes	yes	yes	yes	yes	yes	yes	yes	no	yes	yes	yes	yes	no	yes	yes	no	no	no	no	no	no	yes	yes	no	unknown	yes	free	closed		proprietary	online	graphical user interface	web browser	2022	https://www.wildtrax.ca/home/resources/ guide/intro.html
WASIS – Wildlife Animal Sound Identification System	https://lis-unicamp.github. io/current-projects/wasis/	all	all	all	no	yes	no	yes	yes	no	yes	no	no	yes	yes	no	yes	no	yes	no	no	no	no	yes	no	no	no	no	no	no	yes	free	open	https://github. com/ leandrotacioli/ WASIS	NA	local	graphical user interface	Windows		included in local software
BirdNET Analyzer	https://birdnet.cornell.edu/	birds	all	all	no	no	no	yes	no	yes	no	no	no	yes	no	no	yes	no	no	yes	yes	no	no	no	no	no	no	no	yes	no	no	free	open	https://github. com/kahst/ BirdNET- Analyzer	CC-BY 4.0	online	graphical user interface	web browser		https://birdnet.cornell.edu/live/
Anabat Insight	https://www.titley-scientific. com/eu/anabat-insight. html?SID=bcsn2rrhsh3519j ve9t0cddtl3	bats	all	terrestrial	no	yes	no	yes	yes	yes	unknown	yes	no	yes	yes	yes	yes	yes	yes	yes	yes	no	unknown	yes	no	no	no	no	no	no	no	commercial	closed		proprietary	local	graphical user interface	Windows		https://www.titley-scientific.com/eu/ downloads/dl/file/id/47/product/0/ anabat_insight_user_manual_v2_1.pdf
BCID	https://batcallid. com/allsoftware.html	bats	eastern USA, Canada, UK	terrestrial	NA	yes	NA	NA	NA	NA	NA	NA	NA	NA	NA	yes	NA	NA	yes	yes	yes	NA	NA	NA	NA	NA	NA	NA	NA	NA	NA	commercial	closed		proprietary	local	graphical user interface	Windows		https://drive.switch.ch/index. php/s/9AQunGin4kP1oJw
BatScope 4	https://www.wsl. ch/en/services-and-products/ software-websites-and-apps/ batscope-4.html	bats	Switzerland	terrestrial	no	yes	no	yes	unknown	unknown	yes	no	no	yes	unknown	yes	yes	yes	yes	yes	yes	yes	no	yes	no	no	no	no	no	no	no	commercial	closed		proprietary	local	graphical user interface	Windows		https://drive.switch.ch/index. php/s/9AQunGin4kP1oJw
BatExplorer 2.1	https://www.batlogger.com/ en/downloads/batexplorer/ software/be_2.1/	bats	Europe, UK	terrestrial	yes	yes	yes	yes	yes	no	yes	no	no	yes	yes	yes	yes	yes	yes	yes	yes	yes	yes	yes	no	no	no	yes	no	no	no	commercial	closed		proprietary	local	graphical user interface	Windows	2022	https://www.elekon.ch/batexplorer2/doc/
bcAdmin, bcAnalyze, batIdent	https://ecoobs.de/produkte/ software/	bats	unknown	terrestrial	no	yes	no	yes	yes	yes	unknown	no	no	yes	yes	yes	yes	yes	yes	yes	yes	yes	unknown	yes	no	no	no	no	no	no	no	commercial and free	closed		proprietary	local	graphical user interface	MacOS		https://ecoobs.de/produkte/ software/bcadmin/
AviaNZ	https://www.avianz.net/	birds, bats	New Zealand	all	no	no	no	no	no	yes	no	yes	no	yes	no	yes	yes	yes	yes	yes	yes	no	no	no	no	no	no	no	no	no	no	free on request	open	https://github. com/smarsland/ AviaNZ	GPL-3	local	graphical user interface	Windows,MacOS, Linux	2021	http://www.avianz.net/index.php/avianz- software/user-manual-2
OpenSoundscape	http://opensoundscape. org/en/latest/index.html	all	all	all	no	no	no	no	no	no	yes	no	yes	yes	yes	yes	no	no	no	no	no	yes	yes	yes	yes	no	no	no	no	no	no	free	open	https://github. com/kitzeslab/ opensoundscape	MIT	package	command line	Phyton	2023	http://opensoundscape.org/en/latest/api/ modules.html#module-opensoundscape. signal_processing

***Server installation.*** ecoSound-web is published in a GitHub repository ^58^ and needs to be installed in a web server to run. Instructions and general information regarding the setup for developers and the production server are included in the README file on GitHub. The ecoSound-web installation for local development (in the developer’s machine) is facilitated by a Docker setup. We provide a set of Docker configuration files that can also aid the server installation, but the final setup should be carried out by the server administrator (or devOps engineer) of the institution. For server installations without Docker, a step-by-step installation guide is provided in the repository.

***Access.*** We run an online instance of ecoSound-web ^59^ where the use cases described below can be reproduced. The website hosts several projects belonging to different research groups. One project hosts public reference collections (i.e., reference audio libraries) for Chiroptera, Primata, and Anura, curated by project managers. Soundscape projects can be created per request. Users can access ecoSound-web (both the existing instance and future installations) via a desktop browser with an internet connection. ecoSound-web works with Windows, Linux, and MacOS operating systems and the most common internet browsers (Firefox, Chrome, Safari), but recordings above 96 kHz cannot be played back in Firefox due to browser limitations.

**Figure 2.**
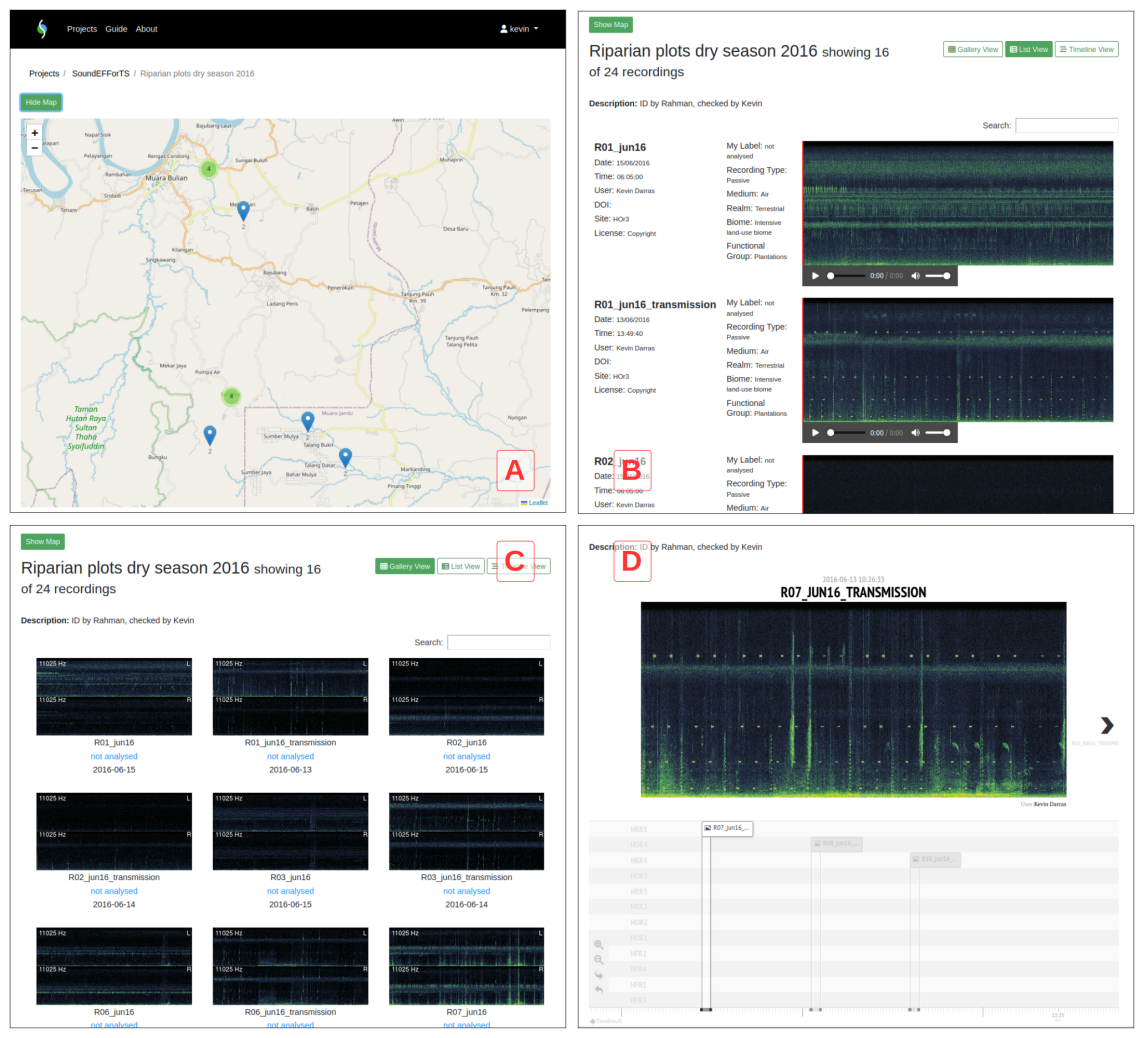
The gallery ( **C**), list ( **B**), and timeline ( **D**) views for recording collections in ecoSound-web, along with the interactive site maps ( **A**).

**Figure 3.**
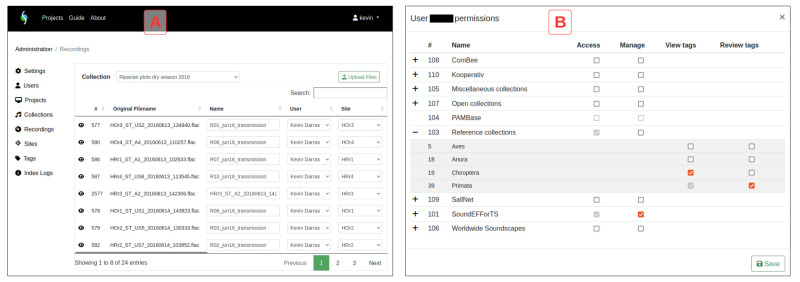
The administrative interface for project managers and administrators. The recording management tab ( **A**), and the user privileges window ( **B**).

***Projects and collections.*** ecoSound-web organises audio recordings within collections, which are grouped under projects. All projects can be accessed through the "Projects" menu, which provides a public overview. Projects and collections are managed in the administrative interface. Projects can contain public (i.e., open) and closed collections, accessible to defined users (c.f. “Users” section). Recordings can be uploaded into collections in most common audio formats ^32^. PNG image previews of the spectrograms and MP3s (for audible sound) or OGGs (for ultrasound > 44100 Hz) of the audio files are generated after insertion into the database, while the original audio files are retained on the server (a download feature is planned). Audio recordings can have custom names to hide the original information present within file names. Collections’ geographic locations are shown on Leaflet-based maps with an OpenStreetMap base layer ( Figure 2A); the recordings listed in the collection are dynamically filtered and clustered based on the current map extent. Collections can be displayed as 1) a list view with larger spectrograms, descriptive data, and a simple audio player, which is particularly suitable for call libraries ( Figure 2B); 2) a simple gallery view displaying spectrogram thumbnails ( Figure 2C); 3) a timeline view where recordings are ordered by sites on the Y axis against a navigable time axis on X ( Figure 2D).

***Users.*** ecoSound-web has two registered user classes with differing privileges: normal users and administrators ( Figure 3B). Administrators have privileges for creating, accessing, editing, and deleting projects, collections, recordings, tags, and users. They can transform users into administrators, or give management privileges to normal users for specific projects so they can act as project managers. Project managers have privileges for creating, accessing, editing, and deleting collections, recordings, tags, and users belonging to their projects. They can give tag view and review privileges to normal users. Normal users have privileges for creating, accessing, editing, and deleting their tags belonging to their collections. If applicable, they can view and review tags of other users as determined by their privileges, and thus act as peer-reviewers.

**Figure 4.**
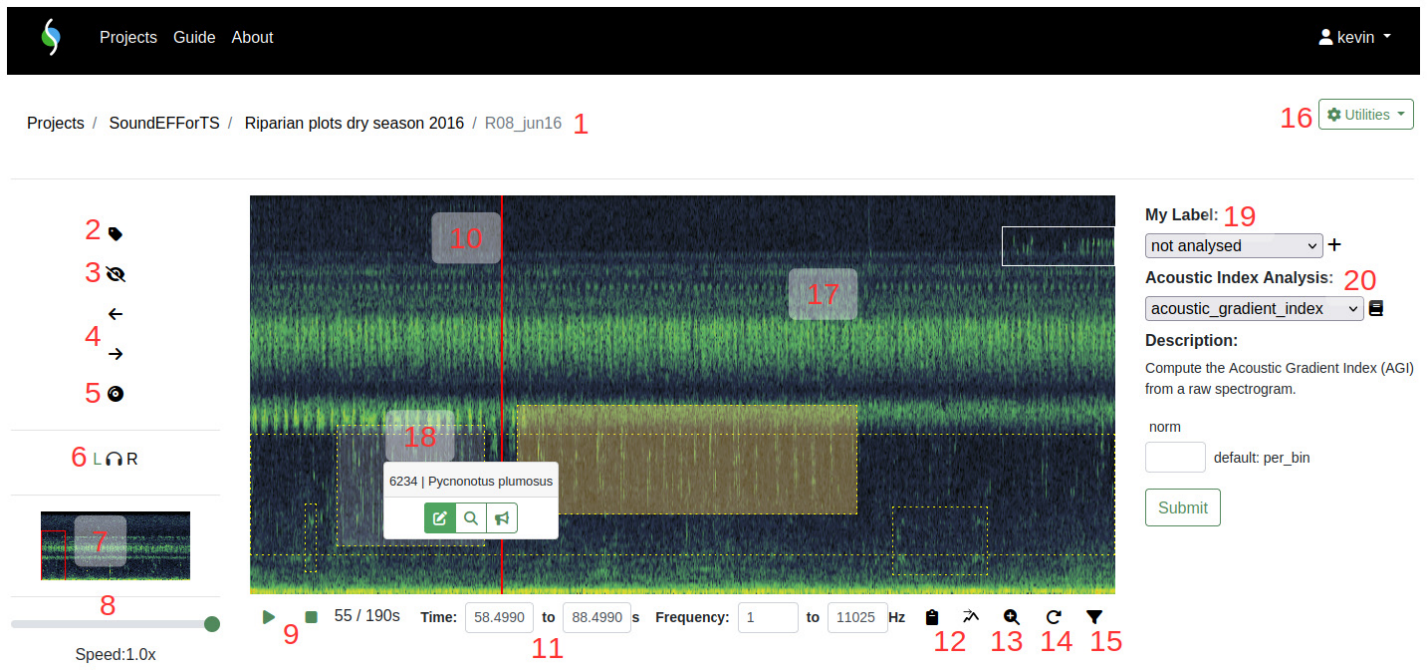
ecoSound-web spectrogram player. Numbers correspond to the following sections: 1: Project/collection/sound name. 2: annotating (i.e., tagging) sounds. 3: hiding/showing tags. 4: panning spectrogram left and right 5: playback mode (zoom to standardised display density). 6: audio channel selection. 7: overview spectrogram, red rectangle shows current view. 8: playback speed slider. 9: playback/pause and stop, time position. 10: playback cursor. 11: time and frequency coordinates of current view or selection. 12: two analysis buttons: copying time and frequency coordinates, exporting frequency of maximum energy. 13: zooming into current selection. 14: continuous playback. 15: frequency filter. 16: utilities menu, containing: image and audio download, FFT window size setting, file info. 17: tags of different users shown with different colors; reviewed tags with solid border, not yet reviewed tags with dashed border; tags without detection distance with orange shading. 18: tag ID and species/sound type appear on click, with buttons for editing, zooming, and estimating distance. 19: assigning pre-set or custom label to recording. 20: computing alpha acoustic indices ^10^.

***Spectrogram player.*** Recordings can be opened in the spectrogram player ( Figure 4). Spectrograms are visualisations of sound where sound amplitude is shown in color or greyscale tones, time is shown on the X axis, and frequency is displayed on the Y axis. Audio channels can be displayed separately. The current spectrogram image and the compressed audio recording (MP3 for audible sound, OGG for ultrasound) can be downloaded. The spectrogram player offers various functionalities for tagging sounds: it is possible to play back sound (at speeds between 0.05 and 1), filter frequencies outside of the current spectrogram view, navigate the spectrogram (zooming per selection, shifting frame sideways, standardized playback at specific display densities), annotate spectrogram regions (creating tags per selection, assigning them to phonies, sound types or soniferous animal species, reviewing and hiding them), label recordings with pre-defined and custom labels, and perform sound analysis (using alpha acoustic indices). Spectrograms are generated after every navigation command, and audio is downloaded on-demand for playback.

## Use cases

### Bird community analysis (Manual annotation and peer-review with different user privileges)

**Figure 5.**
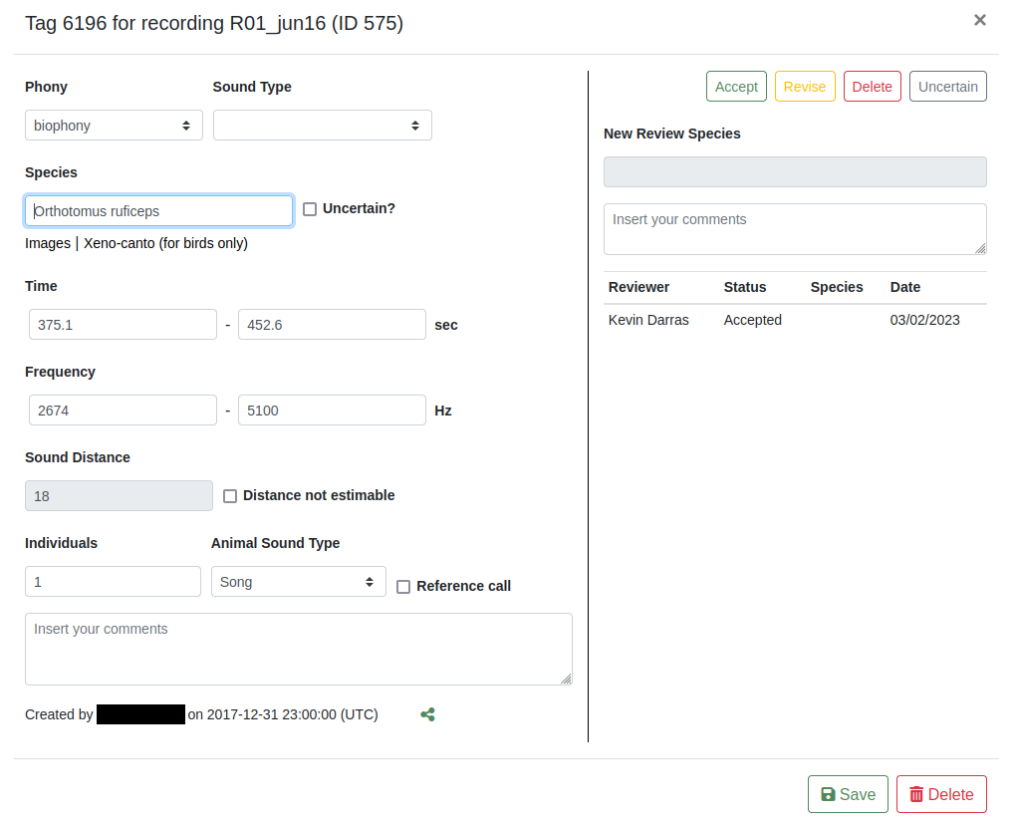
The tag editing window. The phony and sound type selection is remembered for faster tagging. Species, sound distance, individuals, and animal sound type fields are only shown when “biophony” is selected. Sound distance estimation is greyed out because values can only be entered with the dedicated function or declared as not estimable. Green sharing button: copies a URL to the clipboard to open the exact spectrogram region. The entire right pane is only visible to users with reviewing privileges.

Soundscape recordings can be annotated manually for bird vocalisations (or any other sound-producing organisms), and their annotations peer-reviewed by expert ornithologists, as exemplified in the collection " Upland plots dry season 2013". Users can scan recordings visually and aurally using the built-in reading mode, which zooms into the recording to display 15 pixels per second over the entire frequency range (additionally, custom display densities can be set), and enables continuous playback between spectrogram frames. For stereo recordings, the left or right channel can be selected for visually checking vocalisations that may be audible on another channel than the one currently visible. All avian species can be tagged/annotated based on rectangular spectrogram selections along the frequency and time axes. Users can choose species from the integrated species list based on the IUCN Red List taxonomy, and links to Xeno-canto and Google image searches to help the user with identification ( Figure 5). Unclear identifications can be marked as uncertain and additional comments be given. Tags can be designated as reference recordings to be included into future reference collections. Tags can be zoomed into and sharing links can be generated and copied to the clipboard. Any current audio portion can be downloaded as a spectrogram or audio file for sharing with collaborators. Detection distances are estimated by using the distance estimation function ( Figure 4) that enables full-spectrum viewing and playback of the tags based on a spectrogram of the first 30 s of the tag. Additional audio recordings of test tones emitted at known distances are required to help human listeners estimate detection distances in an unbiased way ^60^.

Acoustic recordings can be verified and validated on multiple levels to produce accurate datasets ^61^. In ecoSound-web, tags can be peer-reviewed to validate species identifications and auxiliary data (e.g., distance). Users with reviewing privileges can either accept species identifications, revise them by suggesting other species, mark them as uncertain, or reject them by marking the annotation for deletion ( Figure 5). Administrators can also check the listening durations of each user for each recording to verify that all recordings have been listened to in entirety, and to extract a measure of the sampling intensity. Finally, it is possible to train annotators - after granting them tag viewing privileges - with example annotations of other users. Subsequently, their performance in comparison to already annotated recordings, after revoking tag viewing privileges, can be tested. After the validity checks have been run, users can export the tag data through the administrative interface as a CSV file for further statistical analysis.

### Bat community analysis (working with ultrasound and using the Chiroptera reference collection)

Ultrasonic soundscape recordings can be similarly analysed for bat vocalisations, as shown in the collection " Bat point counts and automated recordings 2019”. However, bat call annotation and analysis present specific challenges. First, bat calls are very short and rapid in succession (units or tens of milliseconds), which is why ecoSound-web generates new spectrograms after zooming, based on precise spectrogram selections. Frequency filtering is enabled by default, so that users hear only what they see on the spectrogram, but can also be disabled. The Fast Fourier Transform (FFT) window size can be set for all recordings accessible to the user (in the administrative interface) and for each recording ( Figure 4) to visualise bat calls better by choosing the ideal trade-off between the frequency and time resolution of the spectrogram. Finally, as ultrasound is not audible, users can adjust playback frequency (continuously between 0.05 and 1) with the playback speed slider to make ultrasound calls audible.

Bat species identification is challenging, as calls from different species can be similar. Additionally to species, custom tag identities can be used for bat sonotypes (i.e., bat call types with similar characteristics across different bat species) ^62^. Exact measurement of bat call features usually determines the assignment of bat calls to specific species. Using the clipboard button ( Figure 4), users can copy the frequency and time coordinates of currently-selected bat calls to derive the start and end frequency, as well as call and call interval duration. Additionally, a dedicated button computes the frequency of maximal energy of the current spectrogram, a metric used for species identification. For species that have taxonomically unequivocal calls, users can refer to the reference collection “ Chiroptera” to corroborate their identifications. As manual distance estimation of bat calls is impractical due to their mobility and the fact that humans cannot intuitively estimate distances of usually inaudible sounds, tags’ distances can be marked as not estimable.

### Primate bioacoustics (working with reference sound libraries)

Reference calls, i.e., example recordings of a animals’ vocalisations, can accelerate the detection of the call (visually or algorithmically) and facilitate the verification of the species identities found in soundscape recordings. Large reference call libraries already exist for birds (Xeno-Canto) and bats (ChiroVox) but are lacking for many other sound-producing animal groups. Available calls from more general libraries such as *tierstimmenarchiv* (" tierstimmenarchiv") contain mostly recordings of captive animals or animals with unknown geolocations, resulting in unclear taxonomies. For primates, acoustic communication has been studied in detail ^63^. However, the potential of passive acoustic monitoring has only recently been acknowledged ^64^ and applied to analyze, e.g., individual caller identity in orangutans ^65^, occupancy in chimps and gibbons ^66, 67^ or density in fork-marked lemurs ^68^, and reference calls have yet to be openly published.

Primate call repertoires range from 7–8 call types in ancestral primates ^69^ to more than 30 individual call types in bonobos ^70^. Many primate vocalizations transmit indexical cues - specific call signatures linked to individuality, sex, population, or species – and they are distributed over a wide range of frequencies extending in the ultrasound field for some basal primates ^69^. This diversity of behavior underlines the importance of their vocalizations ^71^. Although most primate call types are probably used in social contexts over relatively short distances, there is extensive evidence for loud, long-distance calls (several hundreds of meters), that are usually used for intergroup spacing, territorial defense, alarm situations, or as mate advertisement calls ^72, 73^. This provides additional arguments for analysing soundscapes, which can record calls of primates over large areas, to improve future primate population monitoring. However, the lack of publicly available primate reference call libraries slows down its development. Therefore, we initiated the first public primate reference call library based on georeferenced field recordings and annotated vocalisations. Vocalisations are classified into 13 behavioral contexts, such as affiliative, agonistic, contact, or alarm call (see online Guide). The collection is shown in the public “ Reference collection Primata”. DOIs of the respective publication can be assigned to the reference recordings, and Creative Commons licences be chosen to describe usage rights. Distance estimation and collaborative tagging can be used as described above.

### Analysing soundscapes holistically (phonies and acoustic indices)

Soundscapes contain sounds of organismal, human, and geophysical origin ^3^, and their acoustic diversity correlates with biodiversity ^74^. ecoSound-web allows the annotation of sounds with these three different phonies: biophony (sounds of organismal origin), anthropophony (sounds of human origin), and geophony (sounds of geophysical origin), as well as an “unknown” category. Phony annotation is exemplified by the “ Demonstration soundscapes” collection of the Worldwide Soundscapes project on ecoSound-web. Phonies are at the highest level of the sound typology, and only biophony tags allow the specification of the sound-producing species. Within phonies, sound types can be specified, but currently, no systematic typology of sound types exists, so only pre-defined and custom labels are available. In addition, any currently generated audio portion and channel can be analysed with alpha acoustic indices provided by the integrated python package scikit-maad ^10^. Parameters can be input for each function, or left at their default values. Results can be saved by each user and downloaded as a CSV file from the administrative interface ( Figure 3A) for further analysis.

### Future development

We are continuously expanding the functionality of ecoSound-web. Open-source code is a requirement for future development and maintenance. However, it is not a guarantee for a sustainable project either, as some of the open-source automated sound classification tools compiled by Priyadarshani *et al.* in 2018 ^5^ are currently discontinued. In the near future, we will implement the following functions:

1. TensorFlow-based automated detection and classification of vocalisations
2. Expanding sound pressure level analysis and calibration functions
3. Increasing interoperability by linking ecoSound-web to taxonomic databases and ecoacoustic software tools

In ecoSound-web, we implement best coding practices and use development tools, like Docker, to facilitate developers’ work and help them engage in collaboration. We welcome new collaborators to support the project development who could become co-authors on subsequent versions of this article.

## Conclusion

ecoSound-web can be used to store, organize, visualise, play back, peer-annotate, analyse, and share soundscape recordings, tags, collections, and projects online, publicly or with specific users. The recordings can be analysed collaboratively for quantifying soniferous animal species activities such as birds and bats in ecological studies. Furthermore, phonies can be quantified in time, space, and frequency, and alpha acoustic diversity indices can be computed. ecoSound-web has already been used successfully to analyse bird communities ^75^, to measure bat activities ^76^ and to host reference recordings ^68^. Region- and taxon-specific reference collections can be created, like the anuran and primate call collection that we host ^68, 77^.

The field of ecoacoustics and soundscape ecology requires a software tool that standardises and unifies the management and analysis of acoustic data. Although we present one such tool, in the long run, the field is overrun by a multitude of mushrooming projects with unique advantages, for which we compiled the present overview ( Table 1). The sheer number of tools impairs their discoverability, the standardisation of workflows, and their adoption, which is why we decided to integrate an existing tool for the acoustic index analysis (i.e., scikit-maad), as well as a broad thematic scope to include projects from any region, taxon, or realm. A funding and interest-related partitioning and specialisation of the available research platforms (e.g., WildTrax for terrestrial Canada, OPUS for marine Germany, etc.) may be counteracted by APIs enabling inter-operability, and in the broader sense, the FAIR research principles ^4^. Ultimately such developments would stimulate reproducible, cross-realm, and synthetic research based on passive acoustic monitoring methods, potentially even across the Earth System Sciences, where not only ecological, but also geophysical phenomena are analysed.

## Data Availability

All the recordings referred to here are accessible in open collections without login on our online instance of ecoSound-web: https://ecosound-web.de.

## Software availability

Source code available from: https://github.com/ecomontec/ecoSound-web

**Archived source code at the time of publication:** 10.5281/zenodo.7603400.

**License:** GNU General Public License v3.0 (GPLv3).
