# ecoSound-web: an open-source, online platform for ecoacoustics

**DOI:** 10.12688/f1000research.26369.3

**Published:** 2024-11-08

**Authors:** Kevin F.A. Darras, Noemí Pérez, Liu Dilong, Tara Hanf-Dressler, Matthias Markolf, Thomas C Wanger, Anna F. Cord

**Affiliations:** 1 Computational Landscape Ecology, TU Dresden, Dresden, Sachsen, 01737, Germany; 2 Sustainable Agricultural Systems & Engineering Laboratory, School of Engineering, Westlake University, Hangzhou, 310030, China; 3 Agroecology, University of Göttingen, Göttingen, Niedersachsen, 37077, Germany; 4 EFNO, INRAE, Nogent-sur-Vernisson, 46290, France; 5 Quality Technology Centre, Nanjing Julong Steel Pipe Co. Ltd, Nanjing, 211800, China; 6 IUCN SSC Center for Species Survival Cologne Zoo, Cologne Zoo, Cologne, North Rhine-Westphalia, 50735, Germany; 7 Behavioral Ecology & Sociobiology Unit, German Primate Centre, Göttingen, Niedersachsen, 37077, Germany; 8 Key Laboratory of Coastal Environment and Resources of Zhejiang Province, Westlake University, Hangzhou, China; 9 Agro-Ecological Modeling Group, Institute of Crop Sciences and Resource Conservation, University of Bonn, 53113, Bonn, Germany

**Keywords:** Soundscape, sound analysis, ecoacoustics, passive acoustic monitoring, automated sound recording, autonomous recording units, spectrogram, audio annotation

## Abstract

Passive acoustic monitoring of soundscapes and biodiversity produces vast amounts of audio recordings, but the management and analyses of these raw data present technical challenges. A multitude of software solutions exist, but none can fulfil all purposes required for the management, processing, navigation, and analysis of acoustic data. The field of ecoacoustics needs a software tool that is free, evolving, and accessible. We take a step in that direction and present ecoSound-web: an open-source, online platform for ecoacoustics designed and built by ecologists and software engineers. ecoSound-web can be used for storing, re-sampling, organising, analysing, and sharing soundscape recording or metadata projects. Specifically, it allows manual annotation of soniferous animals and soundscape components, automatic annotation with deep-learning models for all birds and for UK bat species, peer-reviewing annotations, analysing audio in time and frequency dimensions, computing alpha acoustic indices, and providing reference sound libraries for different taxa. We present ecoSound-web’s structure and features, and describe its operation for typical use cases such as sampling bird and bat communities, using a primate call library, and the analysis of soundscape components and acoustic indices. ecoSound-web is available from: https://github.com/ecomontec/ecoSound-web

## Introduction

Passive acoustic monitoring, i.e. PAM is a powerful means for monitoring biodiversity and the environment in the field of ecoacoustics ^1^. The resulting soundscape recordings - comprising all sounds recorded in a sea- or landscape ^2^ - present new opportunities for ecologists. However, they yield huge amounts of data that are challenging to manage ^3^ and to analyse for extracting their ecological information – such as biodiversity, human activities, or geophysical events. Overall, soundscape ecologists require a dedicated tool that allows for such a comprehensive workflow ^4^, and which aligns with FAIR research principles ^5^.

Tools for managing ecoacoustic data still undergo rapid development. Annotations identifying sound sources are increasingly generated with artificial intelligence (i.e., AI) methods based on machine learning ^6^ to forego laborious but still common manual annotation by humans. However, even leading AI networks such as BirdNET ^7^ require expert validation to yield usable results ^8^, underlining the importance of vetted reference recordings found in sound libraries to ascertain the identification of sound sources ^9, 10^. Alternatively or in addition to taxonomic annotation of recordings, soundscapes can be characterised with automatically-computed acoustic indices that can measure spectral and temporal variation, entropy, or complexity, and be linked to biodiversity metrics ^11, 12^. General acoustic feature sets can also be used to detect anomalous sound events in an unsupervised manner ^13^. In marine ecoacoustics, annotating and quantifying the temporal proportion of soundscape components (i.e., sounds of biological, geophysical, and human origin) is well-established ^14^, and the sound pressure levels from calibrated equipment are a common metric for studying noise impacts on biological activity ^15^. Finally, in bioacoustic or ethological studies, but also for the identification of bats ^16^ and soundscape characterisation ^17^, the target sounds need to be analysed further by measuring their properties in the frequency-time-amplitude space ^18^.

At the time of writing and to our knowledge, few or no open-source, online software exists that integrates all these different data processing stages into a consistent, integrated workflow for ecoacoustic data across realms, taxa and regions. Reference sound or call libraries are still scarce for particular species groups ^3, 19^, even though recent advances were made for Orthopterans on Xeno-Canto ^9^ (additionally to well-studied birds), and bats on ChiroVox ^10^. Software that handles audio data needs to be built sustainably to benefit a large user base in the research community and to stimulate research ^20^. While the majority of tools are free, few are online-based, many are specialised on specific taxa, realms or regions, only some are open-source, and most cover only parts of the workflow described below. It is essential to have free tools that all researchers and practitioners can use, irrespective of their budget constraints. Also, only open-source projects, in conjunction with long-term vision and funding ^20^, guarantee that they can be continuously developed to keep up with the pace of technological progress, that they stay accessible over time, and that the actual functions are transparent and replicable. Accessibility, which is essential for international collaboration and verification of ecoacoustic data ^21^, also requires online solutions that are mostly independent of operating systems or commercial software. Finally, solutions that integrate multiple steps of the workflow outlined earlier will be inherently less complex for users, more practical, and more replicable than workflows involving separate, specialised tools. In a nutshell, the field of ecoacoustics requires an open-source, online, comprehensive software tool.

Here, we present ecoSound-web: an open-source online platform for ecoacoustics, designed and built by ecologists and software engineers (for the related GitHub project see: https://github.com/ecomontec/ecoSound-web). Currently, ecoSound-web can be used to 1) upload, re-sample, and organize soundscape recordings and metadata within collections and visualize them on maps and timelines; 2) to manage different user types and their access privileges to collections within dedicated projects; 3) to play back, navigate, and filter their sound and spectrograms; 4) to create manual annotations and execute AI models on recording batches for automated annotation of sound sources (i.e., BirdNET for birds ^7^ and batdetect2 ^22^ for UK bats); 5) to peer-review recording annotations; and 6) to measure sounds and compute acoustic indices. ecoSound-web was forked from BioSounds (c.f. article version 1). We detail the structure and functionality of ecoSound-web in the following and announce our development goals.

## Methods

### Implementation

**Figure 1.**
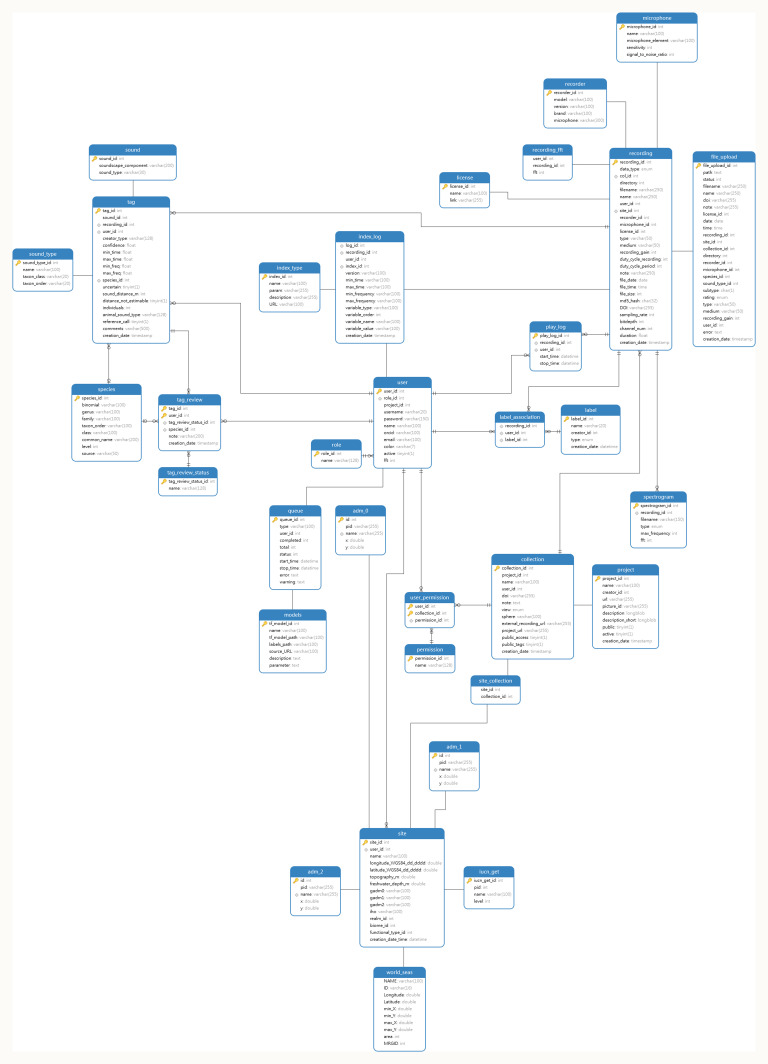
MySQL database structure in ecoSound-web.

***Coding languages, libraries, and tools*.** ecoSound-web is a web-based application written in PHP 7 ^23^, Python 2.7 and 3.10 ^24^, Javascript ^25^, JQuery 3.4 ^26^, Twig 2 ^27^, CSS ^28^ and HTML 5 ^29^. It uses Web Audio API ^30^, Sox 14.4 ^31^, Lame ^32^, ImageMagick ^33^ and Scikit-maad 1.3.12 ^34^ software for sound and image processing, getID3 for audio metadata processing ^35^, a MySQL ^36^ database for organising the data ( Figure 1), a RabbitMQ ^37^ queue for file processing, Plupload 1.5 as a visual file upload tool ^38^, GADM as administrative regions for the sites ^39^, JQuery UI 1.12 ^40^, JCrop 0.9 ^41^, Bootstrap 4.3 ^42^, Leaflet ^43^, Timeline.js ^44^, Bootstrap-selected ^45^, Jquery.cookie ^46^, DataTables ^47^ and the Symfony 4 process component ^48^ for managing the scripts execution. Further Python libraries used are: Numpy ^49^, Pillow ^50^, Audiolab 0.8 ^51^, Matplotlib ^52^, SciPy ^53^, Scikit-image ^54^, TensorFlow ^55^, BirdNET-analyzer ^7^ and batdetect2 ^56^. We containerized the project using Docker ^57^, which spares software developers the time for installing libraries, the database, and configuring the server. This setup allows developers to run the project on their machines quickly and free of typical installation issues like library version incompatibilities.

***Audio visualization and playback*.** The core sound visualisation and playback tasks are handled by two distinct components. First, spectrogram images are generated by the Python script ‘sound visualization tool’, which was created for the discontinued ‘Pumilio’ project ^58^. This script generates spectrograms by computing a Fast Fourier Transform on the waveform of the audio recording, at a user-specified window size (at 128, 256, 512, 1024, 2048, or 4096). Second, sound playback and speed control use Web Audio API, a high-level application programming interface for processing and synthesizing audio in web applications. It is included in modern browsers to take advantage of the browser resources without requiring any extra media player or library in our project.

### Operation

***Ecoacoustic workflow*.** In ecoacoustics, a general workflow is currently not comprehensively defined. We therefore combine insights from different literature sources and our own experience to propose a general workflow as follows: 1) Data management: Acoustic data need to be backed up, archived, and organized according to space, time, and other meta-data ^58^. 2) Signal processing: Recordings can be amplified, re-sampled, split, filtered, compressed, etc. for facilitating the next workflow steps. 3) Audio navigation: Sound recordings can be visualized with spectrograms (i.e., sonograms) or waveforms, navigated in the temporal and spectral dimensions, and played back. 4) Acoustic analysis: Recordings can be annotated with the sound source identity or comments, spectral, temporal, and amplitudinal properties of the recordings can be measured or summarised with acoustic indices or other metrics ^59^. Note that compared to the second version of the present article, we merged the annotation step into the acoustic analysis step and removed the collaboration step, which is not specific to an ecoacoustic workflow. In the following we introduce ecoSound-web to enable the ecoacoustic community to follow and use most parts of the workflow introduced here.

***Installation.*** The ecoSound-web code is published in a GitHub repository ^60^. It needs to be installed in a web server or locally to run. Instructions and general information regarding the setup for developers and the production server are included in the README file on GitHub. The ecoSound-web installation for local development (in the developer’s machine) is facilitated by a Docker setup. We provide a set of Docker configuration files that can also aid the server installation, but the final setup should be carried out by the server administrator (or devOps engineer) of the institution. For server installations without Docker, a step-by-step installation guide is provided in the repository’s Wiki pages.

***Online access.*** We run an online instance of ecoSound-web ^61^ where the use cases described below can be reproduced. The website hosts several projects belonging to different research groups. All projects have project managers and most projects hold data from PAM schemes; one project hosts public reference collections (i.e., reference audio libraries) for Chiroptera, Primata, and Anura. Soundscape projects and project managers can be created per request to KD. Users can access ecoSound-web (both the existing instance and future installations) via a desktop browser with an internet connection. ecoSound-web works with Windows, Linux, and MacOS operating systems and the most common internet browsers (Firefox, Chrome, Safari), but recordings above 96 kHz cannot be played back in Firefox due to browser limitations.

**Figure 2.**
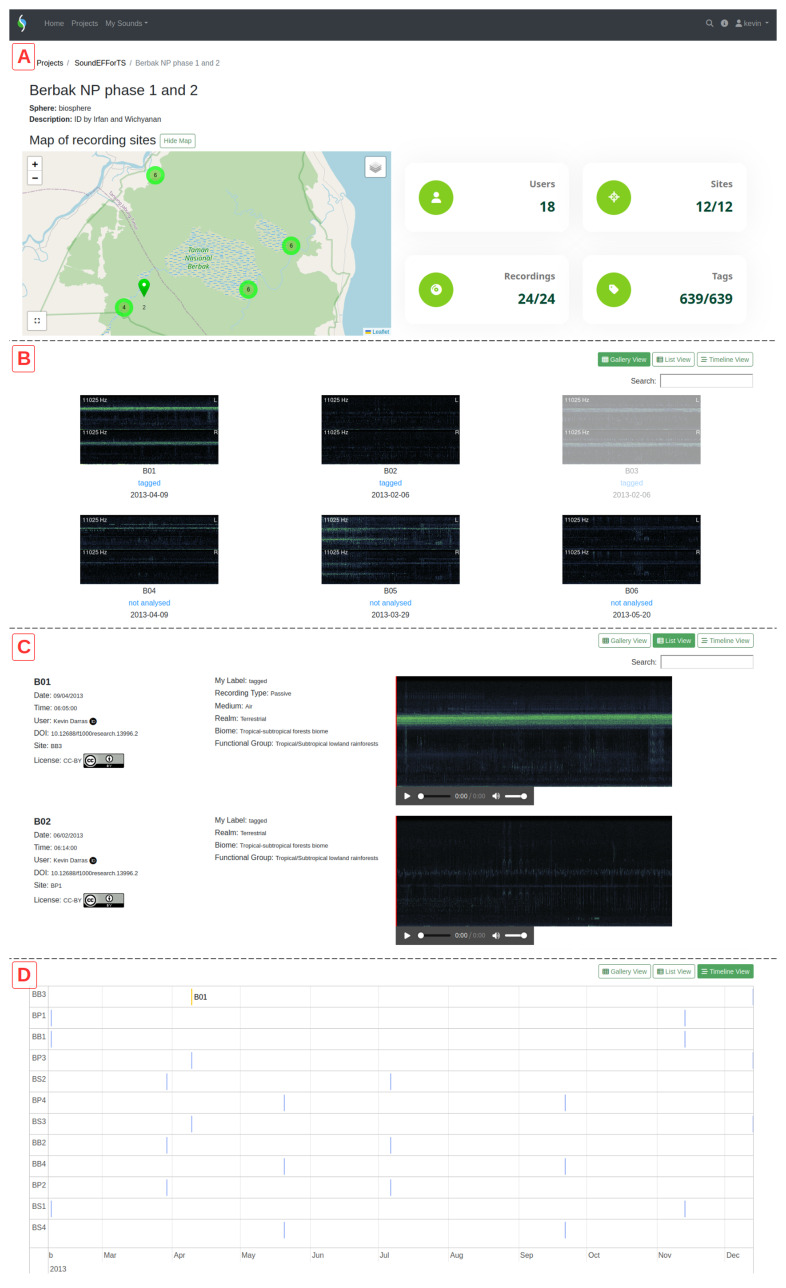
The interactive site maps ( **A**) are shown on top of three available collection views for recordings in ecoSound-web: vignette galleries ( **B**), detailed list with small audio player ( **C**), and navigable timeline ( **D**).

**Figure 3.**
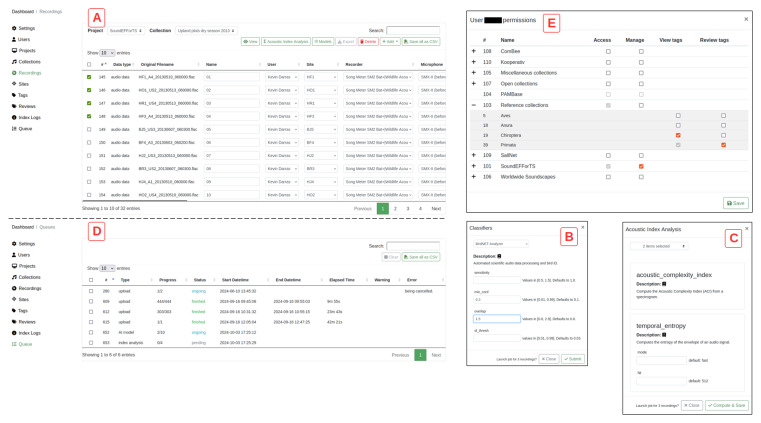
The administrative interface for project managers and administrators. The recordings management tab ( **A**), which allows to run AI models ( **B**) or acoustic index analyses ( **C**) on batches of recordings, whose progress can be checked under the “queue” tab of the dashboard ( **D**). User privileges window visible to administrators and project managers ( **E**).

***Projects and collections.*** ecoSound-web organises audio recordings within collections, which are grouped under projects. All projects can be accessed through the "Projects" menu, which provides a public overview. Projects and collections are managed in the dashboard (previously “administrative interface”). Projects can contain public (i.e., open) and closed collections accessible only to defined users (c.f. “Users” section). Recordings can be uploaded into collections in most common audio formats ^31^. Users can choose to downsample audio recordings during the upload stage. PNG image previews of the spectrograms and MP3s (for audible sound) or OGGs (for ultrasound > 44100 Hz) of the audio files are generated after insertion into the database and the progress of batch insertions of files is shown in the queue tab in the dashboard. The original audio files (or resampled files, if applicable) are retained on the server. Audio recordings can have custom names to hide the original information present within file names. Collections’ geographic locations (“sites” hereafter) are shown on Leaflet-based maps with an OpenStreetMap base layer ( Figure 2A); the recordings listed in the collection are dynamically filtered and clustered based on the current map extent, and the map can be filtered by ecosystem type (as defined by the IUCN Global Ecosystem Typology ^62^). Collections can be displayed as 1) a simple gallery view displaying spectrogram thumbnails ( Figure 2B); 2) a list view with larger spectrograms, descriptive data, and a simple audio player, which is particularly suitable for call libraries ( Figure 2C); 3) a navigable timeline view where recordings are ordered by sites on the Y axis against a time axis on X that can be zoomed and panned ( Figure 2D).

***Recordings.*** Recordings can be managed from the dashboard ( Figure 3A). The original files can be downloaded individually. Their metadata are visible and editable in a table, and the read-only audio tag information of the corresponding files are displayed there (currently supporting ID3Tag, Vorbis, RIFF formats). Recordings are assigned to sites determined by their latitude, longitude, topography (both above and below sea level), depth below the water surface (for freshwater bodies) and ecosystem type. Acoustic index or AI models can be executed on batches of recordings from the dashboard, and the progress of these “jobs” can be checked in the queue tab of the dashboard, where warnings and errors are logged, and jobs can be cancelled and cleared after execution. Meta-recordings can also be integrated in batches with a CSV upload tool. Meta-recordings consist of metadata describing when and where acoustic sampling has been conducted, and with which audio settings (sample rate, channels number, bit depth, recorder and microphone). In contrast to real audio recordings, duty cycles (e.g., 1 minute on, 9 minutes off) can be specified and displayed on the interactive timelines. Large meta-recording collections have been created to provide a global overview of PAM (i.e., Worldwide Soundscapes ^63^).

***Users.*** ecoSound-web has two registered user classes with differing privileges: normal users and administrators ( Figure 3E). Administrators currently include the project lead and web developers; they have privileges for creating, accessing, editing, and deleting projects, collections, recordings, tags, reviews, and acoustic index logs, and disabling users. They can transform users into administrators, or give management privileges to normal users for specific projects so they can act as project managers. Project managers have privileges for creating, accessing, editing, and deleting collections, recordings, tags, reviews, and acoustic index logs, and disabling users belonging to their projects. They can give tag view and review privileges to normal users. Normal users without project management privileges have privileges for creating, accessing, editing, and deleting their tags belonging to their collections. If applicable, they can view and review tags of other users as determined by their privileges, and thus act as peer-reviewers (i.e., annotation validators).

**Figure 4.**
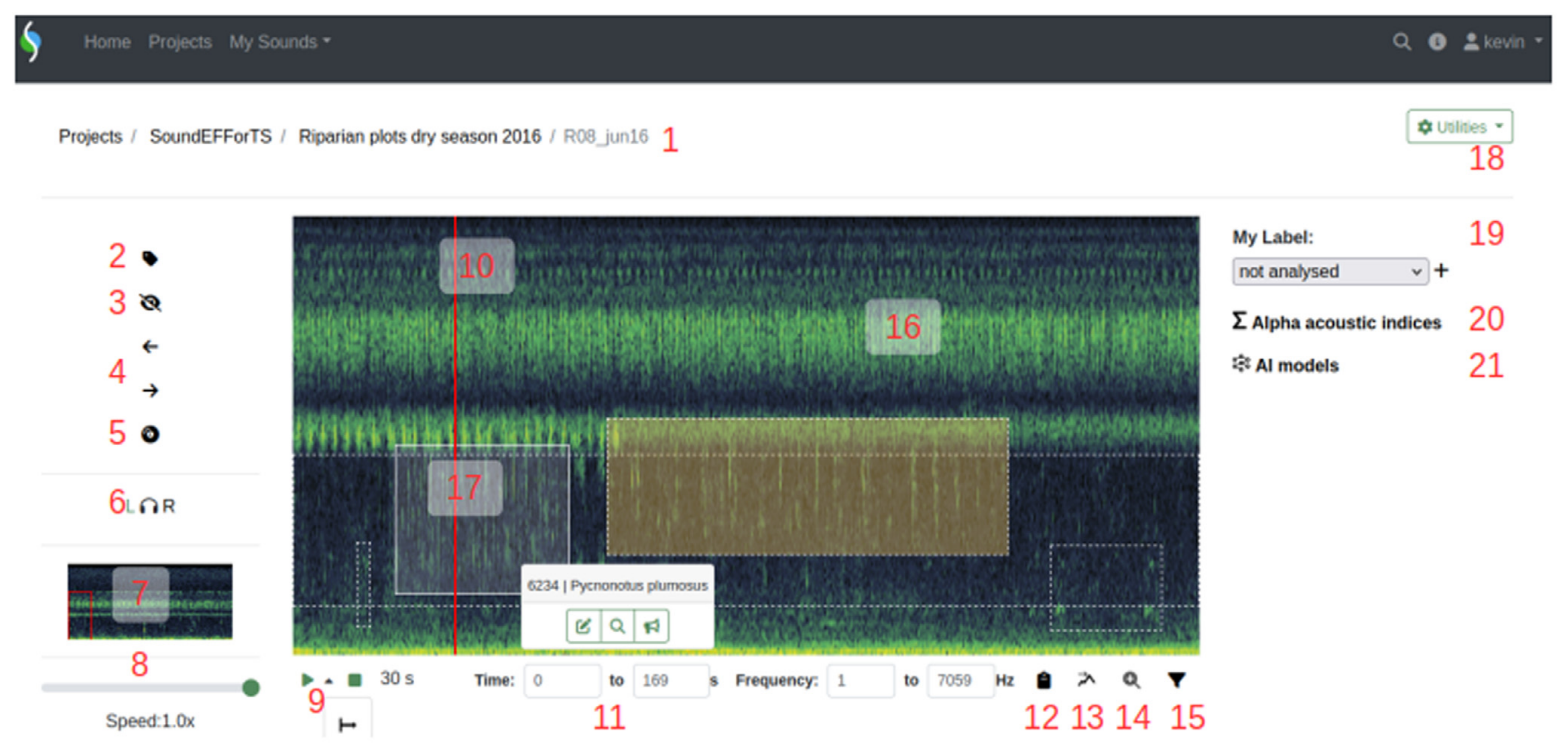
ecoSound-web spectrogram player. Numbers correspond to the following sections: 1: Project/collection/sound name (navigation Breadcrumb). 2: annotate (i.e., tag) sounds. 3: hide/show tags. 4: pan spectrogram left and right 5: playback mode (zoom to pre-defined display density). 6: audio channel selection. 7: overview spectrogram, red rectangle shows current view. 8: playback speed slider. 9: playback/pause (expanded sub-menu allows to activate continuous playback) and stop buttons, time position. 10: playback cursor. 11: time and frequency coordinates of current view or selection. 12: copy time and frequency coordinates to clipboard. 13: export frequency of maximum energy to clipboard. 14: zoom into current selection. 15: frequency filter toggle. 16: tags of different users shown with different colours: reviewed tags with solid border, not yet reviewed tags with dashed border; tags without detection distance with orange shading. 17: tag ID and species/sound type appear on click, with buttons for editing, zooming, and estimating distance. 18: utilities menu, containing: download compressed recording of current view, download original entire recording, download of spectrogram image, file info, recording-level FFT window size settings. 19: assigning pre-set or custom label to recording. 20: computing alpha acoustic indices. 21: executing AI models (BirdNET and batdetect2).

***Spectrogram player.*** Recordings can be opened in the spectrogram player ( Figure 4). Spectrograms are visualisations of sound where sound amplitude is shown in color, time is shown on the X axis, and frequency is displayed linearly on the Y axis. Audio channels can be displayed separately. The current spectrogram image and the compressed audio recording (MP3 for audible sound, OGG for ultrasound) as well as the original recording can be downloaded. The spectrogram player offers various functionalities for tagging sounds: it is possible to play back sound (at speeds between 0.05 and 1), filter frequencies outside of the current spectrogram view, navigate the spectrogram (zooming per selection, shifting frame sideways, playback at specific display densities), annotate spectrogram regions (creating tags per selection, assigning them to soundscape components, sound types or soniferous animal species, reviewing and hiding them), label recordings with pre-defined and custom labels and perform sound analysis using alpha acoustic indices and AI models such as BirdNET and batdetect2. batdetect2 was chosen as it performed well in comparison with other bat call detection and classification AI models and it was easily integrated as a python-based algorithm. The execution parameters of the AI models can be set in the user dialogue and their output is saved as new annotations. Spectrograms are generated after every navigation command, and audio is downloaded on-demand for playback.

## Use cases

### Bird community analysis (Manual and automated annotation, expert peer-review)

**Figure 5.**
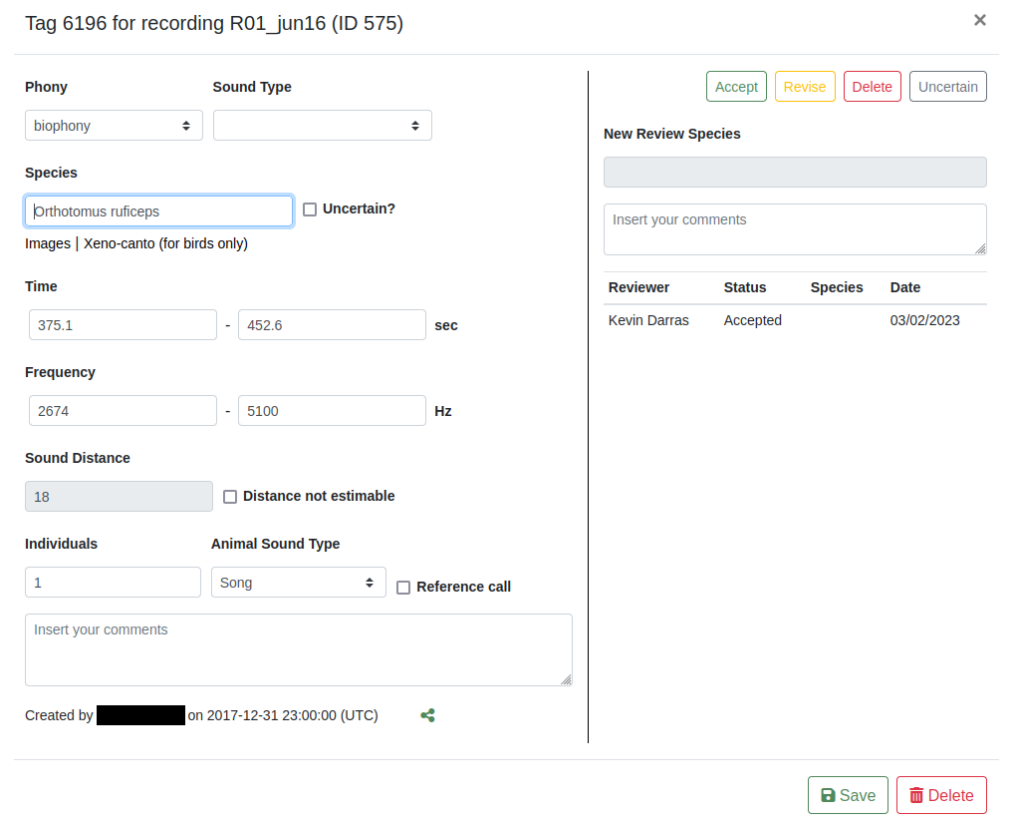
The tag editing window. The soundscape component (currently “phony”) and sound type selection is remembered for faster tagging. Species, sound distance, individuals, and animal sound type fields are only shown when “biophony” is selected. Sound distance estimation is greyed out because values can only be entered with the dedicated function or declared as not estimable. Green sharing button: copies a URL to the clipboard to open the exact spectrogram region. The entire right pane is only visible to users with reviewing privileges. When applicable, the AI model name is displayed after “Creator type” and the confidence score of the detection shown next to it.

Soundscape recordings can be annotated manually or automatically for bird vocalisations, and their annotations peer-reviewed by expert ornithologists, as exemplified in the collection " Upland plots dry season 2013". Manual annotation of any other sound-producing organism is also possible. Users can scan recordings visually and aurally using the built-in reading mode, which zooms into the recording to display 15 pixels per second over the entire frequency range (additionally, custom display densities can be set), and enables continuous playback between spectrogram frames until the recording end. For stereo recordings, the left or right channel can be selected for visually checking vocalisations that may be audible on another channel than the one currently visible. All avian species can be tagged/annotated based on rectangular spectrogram selections along the frequency and time axes. Users can choose species from the integrated species list based on the IUCN Red List taxonomy, and links to Xeno-canto and Google image searches to help the user with identification ( Figure 5). Unclear identifications can be marked as uncertain and additional comments be given. Tags can be designated as reference recordings to be included into future reference collections.

Automated bird identification with BirdNET can be run on any recording (or recordings batch, from the dashboard). The spatial coordinates of recording site are automatically used for filtering BirdNET results, while other execution parameters remain at their default values unless modified by the user ( Figure 3B, custom species list input not yet supported). BirdNET execution will result in the creation of 3-second tags over the entire frequency range, which can subsequently be reviewed by experts. Tags can be zoomed into and sharing links can be generated and copied to the clipboard. Any current audio portion can be downloaded as a spectrogram or audio file for sharing with collaborators. Detection distances can be estimated by using the distance estimation function ( Figure 4): this will execute full-spectrum viewing and playback of the 30 first seconds of the tags; additional audio recordings of test tones emitted at known distances are required to help human listeners calibrate themselves to estimate detection distances in an unbiased way ^64^.

Acoustic recordings can be verified and validated at multiple levels to produce accurate datasets ^65^. Tags can be peer-reviewed to validate species identifications and auxiliary data (e.g., distance). Users with reviewing privileges can either accept species identifications, revise them by suggesting other species, mark them as uncertain, or reject them ( Figure 5). Administrators can also check the listening durations of each user (in the MySQL database) for each recording to verify that all recordings have been listened to in entirety, and to extract a measure of the sampling intensity. Finally, it is possible to train annotators - after granting them tag viewing privileges - with example annotations of other users. Subsequently, their performance in comparison to already annotated recordings, after revoking tag viewing privileges, can be tested. After these validity checks, the tag data can be exported from the dashboard as a CSV file for further statistical analysis.

### Bat community analysis (Manual and automated ultrasound annotation, Chiroptera reference collection)

Ultrasonic soundscape recordings can be analysed for bat vocalisations, as shown in the collection " Bat point counts and automated recordings 2019”. However, bat call annotation and analysis present specific challenges. First, bat calls are very short and occur in rapid succession (milliseconds). Because of this, ecoSound-web generates new spectrograms after zooming, based on the spectrogram selections. Frequency filtering is enabled by default (but can be disabled), so that users hear only what they see on the spectrogram, but it can also be disabled. The Fast Fourier Transform (FFT) window size can be set for all recordings accessible to the user (in the dashboard) and for each recording ( Figure 4) to visualise bat calls better by choosing the ideal trade-off between the frequency and time resolution of the spectrogram. Finally, as ultrasound is not audible, users can adjust playback frequency (continuously between 0.05 and 1) with the playback speed slider to make ultrasound calls artificially audible (analogous to time expansion).

Bat species identification is challenging, as calls from different species can be similar. Additionally to species, custom tag identities can be used for bat sonotypes (i.e., bat call types with similar characteristics across different bat species) ^66^. Exact measurement of bat call features usually determines the assignment of bat calls to specific species. Using the clipboard button ( Figure 4), users can copy the frequency and time coordinates of currently-selected bat calls to derive the start and end frequency, as well as call and call interval duration. Additionally, a dedicated button computes the frequency of maximal energy of the current spectrogram, a metric used for species identification (usually abbreviated “FmaxE”, Figure 4). For uncertain identifications, users can refer to reference collections such as the open “Chiroptera” collection (currently covering selected Southeast Asian species) to corroborate their identifications. As manual distance estimation of bat calls is impractical due to their mobility and the fact that humans cannot intuitively estimate distances of usually inaudible sounds, tags’ distances can be marked as “not estimable”. For automated identification of UK bat species, batdetect2 can be run on any recording (or recordings batch, from the dashboard). The detection threshold stays at its default value unless changed by the user. Upon execution, batdetect2 generates precise tags covering each individually-detected call in the frequency and time dimensions, which can then be reviewed by experts.

### Primate bioacoustics (working with reference sound libraries)

Reference calls, i.e., example recordings of a animal vocalisations, can accelerate the detection of the call (visually or using AI) and facilitate the verification of the species identities found in soundscape recordings. Large reference call libraries already exist for birds (Xeno-Canto) and bats (ChiroVox) but are lacking for many other sound-producing animal groups. Available calls from more general libraries such as tierstimmenarchiv ("tierstimmenarchiv") contain mostly recordings of captive animals or animals with unknown geographic locations, resulting in unclear taxonomies. For primates, acoustic communication has been studied in detail ^67^. However, the potential of PAM has only recently been acknowledged ^68^ and applied to analyze, e.g., individual caller identity in orangutans ^69^, occupancy in chimps and gibbons or howler monkeys ^70– 72^ or density in fork-marked lemurs ^73^, and reference calls have yet to be openly published.

Primate call repertoires range from 7–8 call types in ancestral primates ^74^ to more than 30 individual call types in bonobos ^75^. Many primate vocalizations transmit indexical cues - specific call signatures linked to individuality, sex, population, or species – and they are distributed over a wide range of frequencies extending in the ultrasound field for some basal primates ^74^. This diversity of behaviour underlines the importance of their vocalizations ^76^. Although most primate call types are probably used in social contexts over relatively short distances, there is extensive evidence for loud, long-distance calls (several hundreds of meters), that are usually used for intergroup spacing, territorial defence, alarm situations, or as mate advertisement calls ^77, 78^. This provides additional arguments for analysing soundscapes, which can record calls of primates over large areas, to improve future primate population monitoring. Therefore, we initiated the first public primate reference call library based on georeferenced field recordings and annotated vocalisations. Each recording is assigned to a selectable Creative Commons license and attributed to its recordist. The collection currently contains 76 reference calls of 32 primate species from Indonesia, Myanmar, Madagascar and Peru. Vocalisations are classified into 13 behavioural contexts, such as affiliative, agonistic, contact, or alarm call (see online Wiki). The collection is shown in the public “ Reference collection Primata” and is open for contributions. DOIs of the respective publication can be assigned to the reference recordings. Distance estimation and collaborative tagging can be used as described above.

### Soundscape analyses (soundscape components and acoustic indices)

Beyond particular animal species, soundscapes have components that inform us about organismal, human, and geophysical events ^4^, and their diversity can be measured with acoustic indices ^11^. ecoSound-web hosts the Worldwide Soundscapes project ^63^, a global inventory of PAM conducted in all IUCN realms (i.e., terrestrial, marine, freshwater, and subterranean). Recording sites were assigned to specific ecosystem types, and the interactive sampling site maps can be dynamically filtered to show particular ecosystem selections on three levels (realm, biome, functional group), thereby allowing to identify sampling gaps. Recordings of the project’s demonstration collection, containing samples from different realms, were annotated with three different soundscape components: biophony (sounds of organismal origin), anthropophony (sounds of human origin), and geophony (sounds of geophysical origin) and validated by their recordists using the tag peer-review functionality. The soundscape occupancy (i.e., the occupied proportion of the spectro-temporal space) was thereby analysed after exporting the validated tags’ data as CSV in order to analyse macroecological biophony trends. Soundscape components are the highest level of the sound typology, and sound types can be specified within, using custom labels. However, no systematic typology of sound types currently exists. Furthermore, any currently generated audio portion and channel, and any batch of recordings selected in the dashboard, can be analysed with alpha acoustic indices — which in some cases correlates with biodiversity ^11^ — provided by the integrated python package scikit-maad ^59^. Parameters can be input for each function or left at their default values and multiple indices can be chosen for batch analysis jobs ( Figure 3C). Results can be saved by each user and downloaded as a CSV file from the dashboard’s “index logs” tab ( Figure 3D) for further analysis of the acoustic diversity.

### Future development

We are continuously expanding the functionality of ecoSound-web. Open-source code is a requirement for future development and maintenance, but it is not a guarantee either, so we welcome new collaborators to support the project development who could become co-authors on subsequent versions of this article. In the future, we plan to implement the following functions:

**1.**  Expanding acoustic analysis functions by enabling sound pressure level calibration, facilitating sound detection space measurement, and providing end-to-end validation of AI models
**2.**  Increasing interoperability by linking ecoSound-web to taxonomic databases, external storage, and other software tools

## Conclusion

ecoSound-web can be used to store, re-sample, organize, visualise, play back, peer-annotate, analyse, and share soundscape recordings, tags, collections, and projects online, publicly or with specific users. The recordings can be analysed collaboratively for quantifying soniferous animal species activities (currently birds and bats). Furthermore, soundscape components can be quantified in time, space, and frequency, alpha acoustic diversity indices can be computed, and AI models can be run on recording batches. ecoSound-web has already been used successfully to analyse bird communities ^79^, to measure bat activities ^80^, to host reference recordings ^73^, and to share global meta-recording collections ^63^. Region- and taxon-specific reference collections can be created, like the anuran and primate call collection that we host ^73, 81^.

We acknowledge the necessity of having a diversity of software tools that fulfill the varied needs of users. However, the fields of ecoacoustics and soundscape ecology require a software tool that standardises and unifies the management and analysis of acoustic data. We propose one such tool, but the field is overrun by a multitude of mushrooming software tools with unique advantages (see Table 1 in version 2), and their sheer number impairs discoverability, standardisation of workflows, and adoption. This is why we decided to integrate existing tools for acoustic index analysis (i.e., scikit-maad) and bird and bat identification (BirdNET and batdetect2), and chose a broad thematic scope to include projects from any region, taxon, or realm. A funding- and interest-related partitioning and specialisation of the available research platforms (e.g., WildTrax for terrestrial Canada, OPUS for marine Germany, etc.) may be counteracted by APIs enabling inter-operability, and in the broader sense, the FAIR research principles ^5^. Ultimately, such developments will stimulate reproducible, cross-realm, and synthetic research based on PAM, potentially even across the Earth System Sciences, where not only ecological, but also geophysical phenomena are analysed.

## Data Availability

All the recordings referred to here are accessible in open collections without login on our online instance of ecoSound-web: https://ecosound-web.de.

## Software availability

Source code available from: https://github.com/ecomontec/ecoSound-web

Archived source code at the time of publication: 10.5281/zenodo.13889948.

License: GNU General Public License v3.0 (GPLv3).
