# Supplementary material for: ecoSound-web: an open-source, online platform for ecoacoustics
Source: F1000Res. 2024 Nov 8;9:1224. Originally published 2020 Oct 12. [Version 3] doi: 10.12688/f1000research.26369.3 (PMC7682500; doi:10.12688/f1000research.26369.3)
Supplement: Peer review history

# Reviewer report: Reviewer response for version 3

**DOI:** 10.5256/f1000research.173022.r349021

**Published:** 2025-02-07

**Authors:** Santiago Martinez Balvanera

**Affiliations:** 1 University College London, London, UK

The article presents a web-based platform for eco-acoustic designed to integrate and standardise ecoacoustic workflows.

 I commend the authors for the great work so far on such an important and ambitious project, which clearly involved considerable work and expertise.

 I see the potential value of the platform presented; however, I have a few suggestions that I believe would strengthen both the manuscript and the platform itself.

 While the article is generally well-written and effectively describes the platform's core features, the manuscript would benefit significantly from a clearer and detailed description of the specific gap this platform aims to fill within the field of ecoacoustics.

 A more focused discussion of the need for such a platform, along with a clear explanation of how its features and design address this need, would help the readers understand the platform's purpose and value.

 Specific comments are provided below:

 ## Comments on the Manuscript

 - The manuscript would benefit from clarifying which technical challenges (e.g., storage, processing, analysis) associated with large audio datasets the platform directly addresses.

 - Beyond stating the need for "free, evolving, and accessible" ecoacoustic tools, the manuscript should justify this by detailing the target users and their specific needs that necessitate such a platform.

 - While the manuscript mentions FAIR principles, it would be beneficial to elaborate on what these principles entail and, specifically, how ecoSound-web implements and promotes them.

 - The manuscript should clearly distinguish between human and automated annotation to prevent ambiguity.

   Consider reserving "annotation" for human-generated data and using "prediction" or "detection" for machine outputs, or otherwise ensure a clear separation between the two.

 - I recommend reinstating the Table 1 for clarity and accessibility.

   Its inclusion in previous versions provided valuable context regarding the platform's contribution to the existing ecosystem of tools.

   Referencing earlier versions of the manuscript, as in "see Table 1 in version 2," is unintuitive and may make the information difficult for readers to access.

 - The abstract's claim that the platform enables automatic annotation for "all birds" appears inaccurate.

   If this refers to BirdNET, its training data includes approximately 6,000 species, while the estimated global bird species count exceeds 11,000.

 - While I agree that standardized and integrated workflows for ecoacoustics are valuable, the manuscript needs to be more specific about what this entails in practice.

   Given the diverse applications of ecoacoustics, each requiring specific methodologies and considerations, a single, universal workflow seems unlikely.

   Also, the field is still evolving, with ongoing research exploring the optimal application and effectiveness of various methods.

   The manuscript would benefit from acknowledging this complexity and perhaps focusing on how the platform addresses specific workflows or challenges within this diverse field.

 - I suggest moving the implementation specifics to the supplementary materials.

   While the full database diagram is useful for developers inspecting the database, its inclusion in the main text may be overwhelming for the general reader.

   Consider simplifying the diagram to highlight only the core tables and attributes relevant to the features discussed in the article.

   Similarly, the list of software dependencies, although valuable for developers, would be better placed in the supplementary material or within the software's readme/documentation.

   Software dependencies and their versions are highly dynamic and may be frequently updated, making the information in the article outdated.

 ## Comments on the Platform

 - I strongly recommend migrating any part of the platform still reliant on Python 2.7 to a supported version.

   Python 2.7 reached its end of life in 2020, meaning it no longer receives updates, including critical security patches.

   This poses significant security risks and could expose the platform to vulnerabilities.

 - The documentation should clarify the proper steps for starting ecoSound-web after the first instantiation with `run.sh`.

   Currently, re-running `run.sh` leads to a "FatalError" on `localhost:8080/index`.

   While re-running `install.sh` resolves this it also deletes previous work, and using docker-compose up appears to be the intended method for subsequent initializations.

 - I was unable to run BirdNET on my Linux laptop.

   In batch mode, the BirdNET job appeared in the queue and completed in 0 seconds, but no detections were generated.

   When attempting to run BirdNET on a single recording, an error message was displayed.

   Batdetect2 ran without any issues.

 - Attempting to update project information after creation via the dashboard resulted in the following error for all editable fields: "SQLSTATE[HY093]: Invalid parameter number: parameter was not defined".

 - When creating a site, please specify the units used for the topography input.

   While I suspect it's meters above sea level (MASL), clarifying this in the interface or documentation would be helpful.

 - When attempting to download the CSV of sites, the dropdown menu presented a search input that immediately defocused and closed the dropdown upon any input, preventing its use.

 - Many of the recordings I work with lack complete metadata regarding their origin (date, time, recorder, gain settings, etc.).

   Is it possible to include these recordings in the platform for annotation and analysis despite the missing metadata? Perhaps a feature to mark certain metadata fields as "unknown" or "not applicable" could be implemented.

 - When uploading multiple recordings simultaneously, it's highly unusual for all files to share the same timestamp.

   While I used the "date and time from filename" option, I couldn't specify the format used in my filenames.

   This resulted in incorrect date and time metadata being applied.

   The platform should allow for specifying the date/time format during bulk uploads.

 - The spectrogram's frequency and time range inputs in the lower right corner do not update the display.

   Although the input values change, the spectrogram remains static.

 - When annotating audio, the "Sound Type" field's select input only displays three options, while the user guide mentions several more possible values.

 - When reviewing an annotation, the "New Review Species" input appears to be malfunctioning.

   When attempting to update a batdetect2 detection, I was unable to enter any species name, including "Myotis myotis." The input consistently returned the error: "Please select a species from the list."

 - When running batdetect2 on sample recordings, predictions were generated correctly.

   However, the predicted species is stored in the unlabeled "comments" field instead of the designated "Species" field.

   Storing the predicted species in the "Species" field would improve data consistency and facilitate easier querying.

Are the conclusions about the tool and its performance adequately supported by the findings presented in the article?

Partly

Is the rationale for developing the new software tool clearly explained?

Partly

Is the description of the software tool technically sound?

Yes

Are sufficient details of the code, methods and analysis (if applicable) provided to allow replication of the software development and its use by others?

Yes

Is sufficient information provided to allow interpretation of the expected output datasets and any results generated using the tool?

Yes

Reviewer Expertise:

Machine Learning for Sound Event Detection in Bioacoustics.

I confirm that I have read this submission and believe that I have an appropriate level of expertise to confirm that it is of an acceptable scientific standard, however I have significant reservations, as outlined above.

# Reviewer report: Reviewer response for version 3

**DOI:** 10.5256/f1000research.173022.r357142

**Published:** 2025-01-25

**Authors:** Cristian Pérez-Granados

**Affiliations:** 1 Centre de Ciència i Tecnologia Forestal de Catalunya, Barcelona, Spain

The manuscript is very well written and have no major comments regarding its content. Likewise, EcoSound looks like a very promising platform, which I really want to try. I have a series of minor issues, which I hope may contribute to improve the manuscript and the updated versions of the platform

 1) Regarding the manuscript, I would like to have a larger background about how the platform can be run by the users on their laptop. It is not very clear by reading the manuscript, which are the steps that a user need to know. I can imagine several readers with little expertise on informatics that may have no idea about how the platform can be installed and run on their computer.

 2) Regarding the analyses on BirdNET-Analyzer, I can read "The spatial coordinates of recording site are automatically used for filtering BirdNET results, while other execution parameters remain at their default values unless modified by the user (Figure 3B, custom species list input not yet supported). But, does it mean that the user cannot modify the coordinates? Ideally, it should be optional to run the analyses with or without the coordinates, like when you run BirdNET-Analyzer on Python or in the GUI. A user would like to test how BirdNET performance varies when introducing or not the coordinates. Likewise, most users may also want to run different species lists based on the week when the recording was made. Is that option already available to do it on a batch process? I guess it should be possible to identify the week of recording with the “meta-recordings” option. Remember, that BirdNET count only four weeks per month, so this needs to be considered. Also important, I think that you should say something about the discrepancies between the taxonomy used on EcoSound (IUCN Red List taxonomy) and the one used on BirdNET (Clements Checklist, the version of 2021), since in some cases the species labelled by a human and predicted by BirdNET may vary just because of that. And to finish with BirdNET, in the text seems clear that BirdNET only works for birds, but this is not true, there are several other species included, especially anurans. Although not mandatory, this is something you may want to modify, see e.g:

 Bota G, et al., 2024 (Ref 1)

 Wood C, et al., 2023 (Ref 2)

 Pérez-Granados C, et al., 2023 (Ref 3)

 Btw, in EcoSound it is possible to run own created algorithms on BirdNET or just the ones included by default? It should be stated, like that BirdNET allow users to train and use their own algorithms, a function not included (yet) on EcoSound (if that is the case).

 3) Regarding the annotation part, I think that would be nice to add, if possible and you consider it important, some automated measures of annotation duration, separation between successive annotations of the same species, etc. This may help to make you some idea about how detailed the annotations are. Likewise, a count on the number of species and vocalization in a recording may also help further research testing acoustic indices and sounds’ diversity or sounds’ density. At some point is also said that the tags are exported as CSV,  but what about adding options to download them in other formats, such as Audacity or Raven Pro. I missed some details regarding which signals’ measurements are automatically made, if just some basic measurements are provided allowing users to download the labels in formats available for other sound software may help.

 Minor:

 When a human validate BirdNET predictions and rename the predictions as true/false and rename them, these annotations are saved somewhere? I think it could be nice if all the predictions made for a certain species (even if coming from different projects) are saved in new folders that might be used for training new algorithms. Project after project you can create your own library of labelled snippets by “just” validating BirdNET predictions.

 Do you have any reference for the following statements? “batdetect2 was chosen as it performed well in comparison with other bat call detection and classification AI models and it was easily integrated as a python-based algorithm."

Are the conclusions about the tool and its performance adequately supported by the findings presented in the article?

Yes

Is the rationale for developing the new software tool clearly explained?

Yes

Is the description of the software tool technically sound?

Yes

Are sufficient details of the code, methods and analysis (if applicable) provided to allow replication of the software development and its use by others?

Yes

Is sufficient information provided to allow interpretation of the expected output datasets and any results generated using the tool?

Yes

Reviewer Expertise:

I work mainly on the use of passive acoustics for biodiversity monitoring, the same are of research than the revised manuscript.

I confirm that I have read this submission and believe that I have an appropriate level of expertise to confirm that it is of an acceptable scientific standard, however I have significant reservations, as outlined above.

## References

[1] : Passive acoustic monitoring and automated detection of the American bullfrog. *Biological Invasions* .2024;26(4) : 10.1007/s10530-023-03244-8 1269-1279 10.1007/s10530-023-03244-8

[2] : Passive acoustic surveys and the BirdNET algorithm reveal detailed spatiotemporal variation in the vocal activity of two anurans. *Bioacoustics* .2023;32(5) : 10.1080/09524622.2023.2211544 532-543 10.1080/09524622.2023.2211544

[3] : Combining two user-friendly machine learning tools increases species detection from acoustic recordings. *Canadian Journal of Zoology* .2024;102(4) : 10.1139/cjz-2023-0154 403-409 10.1139/cjz-2023-0154

# Reviewer report: Reviewer response for version 3

**DOI:** 10.5256/f1000research.173022.r357137

**Published:** 2025-01-22

**Authors:** Dominika Winiarska

**Affiliations:** 1 University of Bialystok, Białystok, Poland

The manuscript describes ecoSound-web, an open-source online platform for storing, organising, and analysing soundscape data manually or automatically. While it is generally well-written, I believe some aspects could be explained in greater detail.

 Manuscript comments:

 Introduction

1. „FAIR research principles”, which are… This should be explained with at least an indication of what FAIR research is, since you mention it twice in the paper.

Use cases

1. “Acoustic recordings can be verified and validated at multiple levels to produce accurate datasets.” What exactly do you consider accurate datasets?

Primate bioacoustics

1. There is a mistake with “a” in the first sentence.
2. Xeno-Canto and ChiroVox – the sentence feels redundant to the one in the introduction.

Conclusion

1. There is a reference to the table, but there is none.

References

1. I believe Xeno-Canto should have the reference URL.

Which version of BirdNET and batdetect2 is the app using? I assume it’s sourced directly from GitHub, so it should be indicated that it is the latest available version so the users know that.

 I installed the app via docker and played with it. At first, I had some trouble finding what needed to be filled in to proceed and where to find the required information. For example, several fields must be filled out to upload the files, while others are required for the analysis. I think they should be mentioned in the paper so the user knows what information they need to proceed, or at least the app could mark mandatory fields with an asterisk to avoid the need to click "save" each time to identify missing information. It made me think that a more detailed description of “what goes where” in the paper would be useful and make the app more accessible.

 App comments:

 I was able to use the acoustic indices successfully but encountered an issue with the BirdNET analyser, probably due to some in-app processing on my side. On the other hand, batdetector worked well and detected bat species, though the species name appeared in the comment column, while the species column indicated that the species was unknown.

 I haven’t found the IUCN-based species list nor the behavioural context grouping of the Primata in the open collection that was mentioned in the paper.

 Perhaps the timezone could be set in the user profile, as time is important in the queue and index log. My times were shifted.

 Overall, the app is very useful, and I believe it could be an alternative to some popular acoustic software. Its great advantage is the ability to share and browse collections by multiple users with annotations, which is essential to enhance collaboration efforts. The manuscript clearly describes the platform’s structure and its capabilities. While some functionalities were limited for me, and the app could use further development, I think that ecoSound-web will be a valuable addition to the bioacoustics field, making sound analysis more accessible to researchers.

Are the conclusions about the tool and its performance adequately supported by the findings presented in the article?

Yes

Is the rationale for developing the new software tool clearly explained?

Yes

Is the description of the software tool technically sound?

Yes

Are sufficient details of the code, methods and analysis (if applicable) provided to allow replication of the software development and its use by others?

Yes

Is sufficient information provided to allow interpretation of the expected output datasets and any results generated using the tool?

Yes

Reviewer Expertise:

bioacoustics, passive acoustic monitoring

I confirm that I have read this submission and believe that I have an appropriate level of expertise to confirm that it is of an acceptable scientific standard.

# Reviewer report: Reviewer response for version 2

**DOI:** 10.5256/f1000research.144078.r214163

**Published:** 2023-10-16

**Authors:** Anthony Truskinger

**Affiliations:** 1 Queensland University of Technology, Brisbane, Australia

The authors present ecoSound-web, an ecoacoustics data management system. They explain the workflow they are targeting and the various features they've built into the platform. Several features are novel for web-based ecoacoustics software - such as the use of the Web Audio API. The use of modern software stacks - particularly docker - are explained and validate the claims of maintainable software.

 The lineage for the presented software is Pumilio -> BioSounds -> ecoSound-web. No specific reason was given as to why the project was forked from BioSounds - it would be good if that were addressed.

 This reviewer is the primary developer of the Acoustic Workbench software, an open-source white label ecoacoustics data management platform. There are two instances of the platform: Ecosounds (ecosounds.org) and the Australian Acoustic Observatory (https://acousticobservatory.org/). 

 Given this context, the use of the name ecoSound-web is perhaps a poor choice given the existing Ecosounds project.

 The acoustic workbench supports 5 out of 6 of your workflow steps. It is comparable to (though less feature rich than) ecoSound-web. Given the similarities, one wonders why collaboration or convergence with the Acoustic Workbench is not viable. There are certainly valid reasons, but they don't appear to be explained in your article. As pointed out in your conclusion, our field needs more consensus and less repeated effort to make software sustainable.

 Despite that, the article sufficiently explains the software presented, presents an architecture that makes sense and is technically sound, and is easily replicable.

Are the conclusions about the tool and its performance adequately supported by the findings presented in the article?

Yes

Is the rationale for developing the new software tool clearly explained?

Partly

Is the description of the software tool technically sound?

Yes

Are sufficient details of the code, methods and analysis (if applicable) provided to allow replication of the software development and its use by others?

Yes

Is sufficient information provided to allow interpretation of the expected output datasets and any results generated using the tool?

Yes

Reviewer Expertise:

Ecoacoustics software development

I confirm that I have read this submission and believe that I have an appropriate level of expertise to confirm that it is of an acceptable scientific standard.

# Response

**Published:** 2024-10-25

**Authors:** Kevin Darras

**Affiliations:** EFNO, INRAE, Loiret, France

Thank you for critically reviewing our contribution.

 We will choose a new name, hosting server, and non-forked GitHub repository for the upcoming fourth milestone of ecoSound-web, in line with a continued institutional support that we are currently building up.

 We are open for collaboration with ecoacoustic software tools that share the same goals, as discussed separately. We are currently mapping all available tools for an overview that will be published in a dedicated living systematic review. This necessary first step will help us to achieve transparency in this domain and potentially pave the way for convergence. We discovered many more software tools, so Table 1 was removed but kept for reference in the second version of the present paper.

# Reviewer report: Reviewer response for version 1

**DOI:** 10.5256/f1000research.29110.r74280

**Published:** 2020-11-20

**Authors:** Erin M. Bayne

**Affiliations:** 1 Department of Biological Sciences, University of Alberta, Edmonton, AB, Canada

Biosounds is a very important step forward in the field of bioacoustics. Being able to manage large volumes of digital audio files and the various data that result is a large task that requires "big data" tools. Biosounds is a great step in this direction that continues on from past efforts. I greatly appreciate the efforts the authors have taken to continue developing tools that already existed. Building off of those tools and bringing them together into an easily setup system is a big advance.

 A gap in this particular draft of the paper are other similar efforts that do exist. For example, the group I work with has developed Wildtrax ( www.wildtrax.ca) that has been operational for several years in Canada. This online system is used by partner agencies in provincial and federal governments, not for profits, industry, and academia. It has similar processes to Biosounds in the area of bioacoustics while also providing a platform for the storage of digital images taken by remote cameras. Including this effort as part of the comparison seems warranted. The key difference is Wildtrax is a centralized database system with the goal of sharing data amongst users Canada-wide rather than have each group setup their own server etc. It too is in development and has many things in common with the list of things Biosounds would like to see developed. The key difference seems to be that we have centralized the process and focused on users bringing their data to one place rather than managing things individually.

 While the rationale for WildTrax and Biosounds are different, it does raise what I think is a very important point that would help expand this paper. How do we use tools like WildTrax and Biosounds to create international collaborations and data sharing standards and where might this repository lie? This paper is an excellent description of a wonderful tool but how users that download and setup servers, etc., should consider sharing/using data together is underemphasized. By providing this type of tool Biosounds provides the infrastructure to help us start developing this vision but I feel this paper should also include at least a section for how we should discuss something greater through shared centralized repositories that build off initiatives like Biosounds and WildTrax. Whether a country by country nodal structure is an option that should be discussed rather than having individual labs, researchers, or local government departments create their own systems is the next step in determining how we can better utilize the amazing power that autonomous recording devices give us. A discussion of how such a process might be facilitated internationally (i.e. through ornithological societies, bat societies, etc.) would help develop the vision that I think Darras *et al. *and others envision.

 Darras et al must be complemented for developing such a useful tool that will undoubtedly improve the field of bioacoustics immeasurably. It is now up to the bioacoustics and wildlife biology fields to determine the best ways of making this data the most useful for the advancement of science and conservation.

Are the conclusions about the tool and its performance adequately supported by the findings presented in the article?

Yes

Is the rationale for developing the new software tool clearly explained?

Yes

Is the description of the software tool technically sound?

Yes

Are sufficient details of the code, methods and analysis (if applicable) provided to allow replication of the software development and its use by others?

Yes

Is sufficient information provided to allow interpretation of the expected output datasets and any results generated using the tool?

Yes

Reviewer Expertise:

I am working in very similar areas trying to achieve a similar objective. My group uses ARUs for a multitude of objectives and needed a tool that could do what is described here. We took a different path than Biosounds but what they have developed is an excellent resource I can see drawing from.

I confirm that I have read this submission and believe that I have an appropriate level of expertise to confirm that it is of an acceptable scientific standard.

# Response

**Published:** 2023-02-06

**Authors:** Kevin Darras

**Affiliations:** EFNO, INRAE, Loiret, France

Thank you for your very interesting, complementary appreciation of our work. In the meantime, we have deployed a new version, adding project management, acoustic indices, phonies, maps, and timelines to ecoSound-web.

 We have discussed the important points you raised in the conclusion with a new paragraph, and we adapted the goals to emphasize inter-operability. These points, together with the need for standards, are also themes in the focus of the first authors’ current research projects and interests, which will be expanded further in the next article iteration and website version (c.f. GitHub issues). Note that we already implemented DOI referencing and the choice of Creative Commons licenses for the recordings.

# Reviewer report: Reviewer response for version 1

**DOI:** 10.5256/f1000research.29110.r74102

**Published:** 2020-11-10

**Authors:** Sarab S. Sethi

**Affiliations:** 1 Department of Mathematics, Imperial College London, London, UK

In this study Darras *et. al* present Biosounds, an open source web-based platform for archiving, exploring, and tagging eco-acoustic data. The tool fulfills an important role, and provides ecologists with a user friendly, powerful tool for managing data acquired from projects deploying acoustics.

 Whilst the paper was generally well written and clear, my line by line recommendations/comments are as follows:

**Introduction**

- “19 reported software tools by Priyadarshani” consider rephrasing to “19 software tools reviewed by…” to ensure it is clear that these tools weren’t developed by Priyadarshani *et. al.*
- Table 1 presents a comprehensive comparison between Biosounds and other tools. However, it is difficult to parse efficiently in its current format, with a large number of factors to consider, and sorting all the options just alphabetically. Maybe sorting from most to least features, or grouping tools in some way would allow for faster comparisons to be made between the offerings. Additionally, wherever it is placed, putting Biosounds in bold would improve the table.
- Table 2, whilst again comprehensive, is arguably not too interesting. Picking out the key few differences and presenting them inline in the text may be a clearer way to present this information – readers are perhaps unlikely to read through the entire page long table. The full table could then be left in supplementary materials

**Methods**

- “by computing a fast fourier transform” – what are the parameters for this? And why were they chosen? Are they customisable / do they adapt based on recording parameters?
- “Soundscape collections, due to their larger size, can be integrated up to a manageable size” – giving an indicative size in GB/TB is crucial for readers to assess whether this tool is suitable for their own projects.
- “upload recordings in most common audio formats” – please detail exactly which formats are supported.
- “MP3s of the audio file are generated after insertion into the database” – are the original uploaded audio files retained on the server (e.g., in the case of raw WAV files being uploaded)? Can the users download these back at a later date, or only MP3s? How are the MP3s generated (which codec) and at which compression level?

**Use-cases**

- “distances are estimated in a standardised way” – how? Using spherical sound absorption assumptions, or by other means? Also, how is distance estimated without knowing the typical source SPL value of a species’ call?
- “export the tag data through a MySQL database” – are users (not administrators) also able to access this data? It would also be useful to add CSV export option for those not so comfortable with SQL DBs.
- “bat morphospecies …. named with single letters from A to J” – you should either detail how these morphospecies differ (what are the identifying call characteristics that places a bat in B for example), or if irrelevant leave out this level of detail and just mention that morphospecies are supported too.

I also had a play around with the live demo running at https://soundefforts.uni-goettingen.de/biosounds/. My overall impression was very positive – the site was intuitive to interact with and things generally worked as advertised. I do have a few very minor recommendations below but appreciate that fulfilling these may be more difficult than the suggested manuscript changes:

- Opening the player view for a large file can take some time, and it just seems as if the connection has dropped (until finally the page is ready). This is not an issue when going forward and backward within the collection as there is a loading overlay, which makes it clear what is happening.
- When loading a large file into the player view the “play” icon is grayed out until some background loading is done. It isn’t clear that this is happening until this is done though – maybe a loading indicator in place of the gray play icon would be clearer.
- Once I’d zoomed in on a part of the spectrogram I couldn’t return to the original, zoomed-out view without refreshing the page. This should be possible.
- When in the player mode there is no indication of sampling frequency or labels on the spectrogram’s Y axis. This info can be inferred from the frequency selection box values, but it should be more clearly apparent in my opinion as it is such an important piece of information. If an expert is searching for calls from a specific species, frequency labels on the Y axis can make this a lot quicker.
- When searching through a collection, if you click on a species from the dropdown prompt, the expected behaviour is that the search will be performed. However, instead a search isn’t actually triggered until you click the search button manually.

Are the conclusions about the tool and its performance adequately supported by the findings presented in the article?

Yes

Is the rationale for developing the new software tool clearly explained?

Yes

Is the description of the software tool technically sound?

Yes

Are sufficient details of the code, methods and analysis (if applicable) provided to allow replication of the software development and its use by others?

Yes

Is sufficient information provided to allow interpretation of the expected output datasets and any results generated using the tool?

Yes

Reviewer Expertise:

eco-acoustics, bioacoustics, ecological monitoring, software development

I confirm that I have read this submission and believe that I have an appropriate level of expertise to confirm that it is of an acceptable scientific standard.

# Response

**Published:** 2023-02-06

**Authors:** Kevin Darras

**Affiliations:** EFNO, INRAE, Loiret, France

Thank you for the detailed appraisal of our work. In the meantime, we have deployed a new version (adding project management, acoustic indices, phonies, maps, timelines) and addressed your comments.

 Introduction: We removed the ambiguous sentence, which was mentioned in the discussion anyway. ecoSound-web is now in bold, and the Table 1 structure follows the classification of purposes announced in the introduction. Table 2 was deleted.

 Methods: We newly implemented FFT window size settings for the users (globally and per recording) and mention the available values in the text. We removed the uninformative “manageable size” and mention the user self-registration feature (which depends on long-term funding) in the conclusion’s goals. Regarding the SoX audio formats: the list is too long so we prefer to refer to the SoX homepage with a citation, but you can find them at https://sox.sourceforge.net/soxformat.html . We now mention that original audio files are retained on the server, but we don’t provide download features yet (see issue #99 on GitHub).

 Use cases: We already provide a citation (10.1111/2041-210X.13031) for the standardised estimation of the detection distances but added some more information to make clear that the reference recordings help human listeners to estimate distances. We now offer CSV export functions for the various data tables (tags, recordings, collections, acoustic indices) that are accessible to users with variable privileges instead of the impractical MySQL access option. Finally, the mention about using letters to distinguish morphospecies (we use “sonotypes” instead) was removed too.

 Regarding general usability comments: Spectrogram computation is still time-consuming as it is computationally demanding, but we implemented on-demand audio loading: the playback button can be clicked, it will become gray while the audio is loaded, and start playback and transform into a pause button. This avoids downloading large files for users who are only tagging visually (e.g., for bat recordings). We have restored the functionality for returning to the previous spectrogram view with an instant back browser button press. However, we decided to keep the time and frequency indications inside the selection boxes only instead of adding scales, as we find that information redundant and haven’t found a best practice implementation yet (but see issue #162). Finally, our new collection search function instantly searches across all the files’ meta-data and does not require a search button press.
