# Supplementary material for: ecoSound-web: an open-source, online platform for ecoacoustics
Source: F1000Res. 2023 Mar 1;9:1224. Originally published 2020 Oct 12. [Version 2] doi: 10.12688/f1000research.26369.2 (PMC7682500; doi:10.12688/f1000research.26369.2)
Supplement: Peer review history

# Response

**Published:** 2023-02-06

**Authors:** Kevin Darras

**Affiliations:** TU Dresden, Germany

Thank you for your very interesting, complementary appreciation of our work. In the meantime, we have deployed a new version, adding project management, acoustic indices, phonies, maps, and timelines to ecoSound-web.

 We have discussed the important points you raised in the conclusion with a new paragraph, and we adapted the goals to emphasize inter-operability. These points, together with the need for standards, are also themes in the focus of the first authors’ current research projects and interests, which will be expanded further in the next article iteration and website version (c.f. GitHub issues). Note that we already implemented DOI referencing and the choice of Creative Commons licenses for the recordings.

# Response

**Published:** 2023-02-06

**Authors:** Kevin Darras

**Affiliations:** TU Dresden, Germany

Thank you for the detailed appraisal of our work. In the meantime, we have deployed a new version (adding project management, acoustic indices, phonies, maps, timelines) and addressed your comments.

 Introduction: We removed the ambiguous sentence, which was mentioned in the discussion anyway. ecoSound-web is now in bold, and the Table 1 structure follows the classification of purposes announced in the introduction. Table 2 was deleted.

 Methods: We newly implemented FFT window size settings for the users (globally and per recording) and mention the available values in the text. We removed the uninformative “manageable size” and mention the user self-registration feature (which depends on long-term funding) in the conclusion’s goals. Regarding the SoX audio formats: the list is too long so we prefer to refer to the SoX homepage with a citation, but you can find them at https://sox.sourceforge.net/soxformat.html . We now mention that original audio files are retained on the server, but we don’t provide download features yet (see issue #99 on GitHub).

 Use cases: We already provide a citation (10.1111/2041-210X.13031) for the standardised estimation of the detection distances but added some more information to make clear that the reference recordings help human listeners to estimate distances. We now offer CSV export functions for the various data tables (tags, recordings, collections, acoustic indices) that are accessible to users with variable privileges instead of the impractical MySQL access option. Finally, the mention about using letters to distinguish morphospecies (we use “sonotypes” instead) was removed too.

 Regarding general usability comments: Spectrogram computation is still time-consuming as it is computationally demanding, but we implemented on-demand audio loading: the playback button can be clicked, it will become gray while the audio is loaded, and start playback and transform into a pause button. This avoids downloading large files for users who are only tagging visually (e.g., for bat recordings). We have restored the functionality for returning to the previous spectrogram view with an instant back browser button press. However, we decided to keep the time and frequency indications inside the selection boxes only instead of adding scales, as we find that information redundant and haven’t found a best practice implementation yet (but see issue #162). Finally, our new collection search function instantly searches across all the files’ meta-data and does not require a search button press.
